# The use of inhaled corticosteroids in pediatric asthma: update

**DOI:** 10.1186/s40413-016-0117-0

**Published:** 2016-08-12

**Authors:** Elham Hossny, Nelson Rosario, Bee Wah Lee, Meenu Singh, Dalia El-Ghoneimy, Jian Yi SOH, Peter Le Souef

**Affiliations:** 1 Pediatric Allergy and Immunology Unit, Children’s Hospital, Ain Shams University, Cairo, 11566 Egypt; 2 Federal University of Parana, Curitiba, Brazil; 3 Khoo Teck Puat-National University Children’s Medical Institute, National University Health System, Singapore, Singapore; 4 Department of Paediatrics, Yong Loo Lin School of Medicine, National University of Singapore, Singapore, Singapore; 5 Postgraduate Institute of Medical Education and Research, Chandigarh, India; 6 Winthrop Professor of Paediatrics & Child Health, School of Paediatrics & Child Health, University of Western Australia, Crawley, Australia

**Keywords:** Inhaled corticosteroids, Children, Asthma, Adverse effects, Adherence

## Abstract

Despite the availability of several formulations of inhaled corticosteroids (ICS) and delivery devices for treatment of childhood asthma and despite the development of evidence-based guidelines, childhood asthma control remains suboptimal. Improving uptake of asthma management plans, both by families and practitioners, is needed. Adherence to daily ICS therapy is a key determinant of asthma control and this mandates that asthma education follow a repetitive pattern and involve literal explanation and physical demonstration of the optimal use of inhaler devices. The potential adverse effects of ICS need to be weighed against the benefit of these drugs to control persistent asthma especially that its safety profile is markedly better than oral glucocorticoids. This article reviews the key mechanisms of inhaled corticosteroid action; recommendations on dosage and therapeutic regimens; potential optimization of effectiveness by addressing inhaler technique and adherence to therapy; and updated knowledge on the real magnitude of adverse events.

## Background

Despite advances in care, asthma still imposes a significant burden on the pediatric population. Mortality, hospitalization rates, acute exacerbations and symptom control remain sub-optimal. In controlled trials, most patients gain high levels of control but in ‘real-life’ clinical practice, most patients do not [1]. Communication between clinicians and patients is sometimes poor, and it should be noted that in low income countries, an important obstacle to proper asthma management is the cost of medications [2].

Inhalation therapy is the cornerstone of asthma treatment in the pediatric age group [3]. The concern about adverse effects induced by systemic CS in children has been much reduced, but not eliminated, with the use of the inhalation route [4]. In this document, the current literature concerning strategies to improve outcome from inhaled corticosteroid (ICS) use is reviewed with the goal of highlighting the potentials and pitfalls encountered.

## Mechanisms of action of ICS

Inhaled corticosteroids are the most effective drugs used in asthma to suppress airway inflammation. This occurs mainly by down regulation of pro-inflammatory proteins [5, 6]. Also, corticosteroids seem to reverse components of the asthma-induced structural changes (airway remodeling), including the increased vascularity of the bronchial wall [7].

At a cellular level, ICS reduce the number of inflammatory cells in the airways, including eosinophils, T lymphocytes, mast cells, and dendritic cells. These remarkable effects of corticosteroids are produced by suppressing the production of chemotactic mediators and adhesion molecules and by inhibiting the survival of these inflammatory cells in the airways. Epithelial cells may be a major cellular target for ICS, which are the mainstay of modern asthma management [8].

The broad anti-inflammatory profile of corticosteroids probably accounts for their marked clinical effectiveness in asthma [9]. More specific treatment options such as single mediator inhibitors have usually been unsuccessful, emphasizing the importance of simultaneously inhibiting many inflammatory targets [10].

**Fig. 1:**
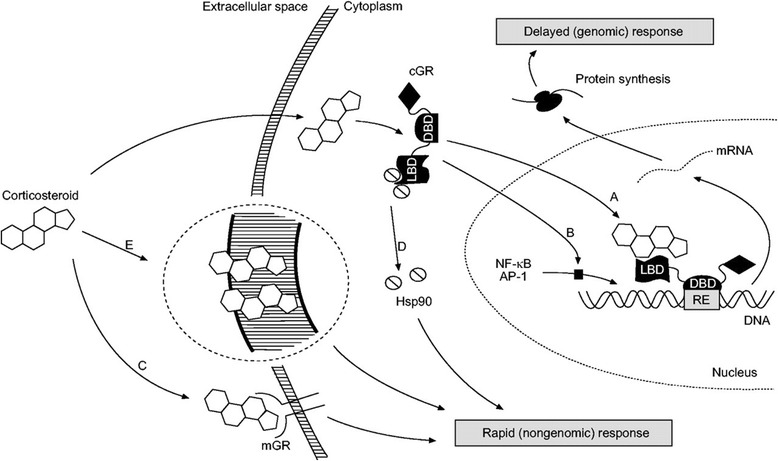
Schematic diagram of the complex cellular actions of corticosteroids. Genomic actions are mediated by cytoplasmic receptors, which ultimately alter transcription through A direct DNA binding or B transcription factor inactivation. In contrast, nongenomic actions are mediated by C membrane-bound or D cytoplasmic receptors, or E nonspecific interactions with the cell membrane. cGR: cytoplasmic glucocorticoid receptor; mGR: membrane glucocorticoid receptor; LBD: ligand-binding domain; DBD: DNA-binding domain; Hsp90: heat-shock protein 90; RE: response element; NF-kB: nuclear factor-kB; AP-1: activating protein-1. Quoted with permission from: Horvath, G and Wanner, A. Eur Respir J 2006;27:172–87

The anti-inflammatory actions of ICS are divided into considerably delayed actions (taking hours or days) through genomic mechanisms required to change the protein expression and rapid actions (within seconds or minutes) probably mediated through membrane-bound glucocorticoids receptor and direct interaction with the airways vasculature by non-genomic mechanisms [11]. (Table 1; Fig 1).

**Table 1:** Mechanism of actions of inhaled corticosteroids in asthma

	Genomic	Non-genomic
Action mediated through	Cytoplasmic glucocorticoid receptor-α [9].	Membrane-bound or cytoplasmic glucocorticoid receptor or direct interaction with airway vasculature [11].
Onset of action	Hours to days [11].	Seconds to minutes [11].
Effects	- Selective switch off in multiple activated inflammatory genes (transrepression) by reversal of histone acetylation [9, 15, 16]. - Increasing mRNA degradation and hence blocking production of pro-inflammatory cytokines [11]. - Increasing the synthesis of anti-inflammatory proteins [9].	- Suppressing the increased microvascular permeability and plasma leakage into the airway lumen [29–32]. - Acutely suppressing airway hyperperfusion in a dose-dependent manner [27]. - Inhibiting the remodeling process (only long-term therapy in a dose-dependent manner) [33, 34].

### Genomic mechanism of action of inhaled corticosteroids

These are genomic actions mediated by intracellular receptors; glucocorticoid receptors (GRs), which ultimately alter transcription through direct DNA binding or indirectly through transcription factor inactivation [12].

The lipophilic corticosteroid molecules easily cross the lipid bilayer of the cell membrane to enter into the cell and bind to specific receptors [13]. Glucocorticoid receptor-α binds corticosteroids, whereas glucocorticoid receptor-β is an alternatively spliced form that binds to DNA but is not activated by corticosteroids [9].

#### Direct genomic effects

After corticosteroids have bound to GRs, changes in the receptor structure result in dissociation of molecular chaperone proteins, thereby exposing nuclear localization signals on GRs. This results in rapid transport of the activated glucocorticoid receptor-corticosteroid complex into the nucleus, where it binds to DNA at specific sequences in the promoter region of steroid-responsive genes known as glucocorticoid response elements (GRE) [9]. A pair of GRs (GR homodimer) bind to GRE in the promoter region of steroid-responsive genes and this interaction switches on (and sometimes switches off) gene transcription [14].

The major action of corticosteroids is to switch off multiple activated inflammatory genes that encode for cytokines, chemokines, adhesion molecules inflammatory enzymes and receptors [15]. Repression of genes occurs through reversal of the histone acetylation that switches on inflammatory genes [16]. Activated GRs may bind to cyclic adenosine monophosphate response element–binding protein (CBP) or other co-activators like p300/CBP-activating factor (PCAF) directly to inhibit their histone acetyl transferase (HAT) activity [17], thus reversing the unwinding of DNA around core histones and thereby repressing inflammatory genes. Reduction of histone acetylation also occurs through the recruitment of histone deacetylase-2 (HDAC2) to the activated inflammatory gene complex by activated glucocorticosteroid receptor, thereby resulting in effective suppression of all activated inflammatory genes within the nucleus [14].

Corticosteroids may suppress inflammation by increasing the synthesis of anti-inflammatory proteins, such as annexin-1, secretory leukoprotease inhibitor, interleukin-10, and an inhibitor of nuclear factor-kB (IkB-α) (transactivation) [9] However, this mechanism seems to have a minor role in the suppression of inflammation. For instance, corticosteroids have been reported to repress inflammation efficiently in mice with a defective glucocorticoid receptor, which cannot bind DNA [18]. Also, therapeutic doses of ICS have not been shown to increase annexin-1 concentrations in bronchoalveolar lavage fluid [18]. It has been proposed that transactivation is responsible for some side effects (e.g. diabetes induction, skin atrophy) caused by corticosteroids [19].

#### Indirect genomic effects

Corticosteroids have been shown to have post-transcriptional regulatory effect on gene expression through increasing messenger RNA (mRNA) degradation, and thus block the production of several of pro-inflammatory cytokines and other proteins (transrepression) [20]. Corticosteroids reduce the stability of mRNA for some inflammatory genes, such as cyclooxygenase-2, through an inhibitory action on p38 mitogen-activated protein kinase (p38 MAP kinase) [21]. This inhibitory effect is mediated via the induction of a potent endogenous inhibitor of p38 MAP kinase called MAP kinase phosphatase-1 [22].

GRs probably bind only to coactivators that are activated by pro-inflammatory transcription factors, such as nuclear factor-kB (NF-kB) and activator protein-1 (AP-1). Also, it is likely that several specific coactivators interact with GRs. This might explain why corticosteroids switch off only inflammatory gene and are well tolerated as a therapy [9].

### Non-genomic mechanism of action of inhaled corticosteroids

Non-genomic actions are initiated by specific interactions with membrane-bound or cytoplasmic GRs, or nonspecific interactions with the cell membrane [11]. Membrane-bound GRs are also present in various human cells and peripheral blood mononuclear cells [23]. Also, membrane binding sites for corticosteroids have been demonstrated in smooth muscle cells isolated from human airway blood vessels [24]. Membrane receptor activation has been shown to induce rapid effects on a variety of second messenger systems (Calcium, adenosine 3ʹ, 5ʹ-monophosphate, inositol trisphosphate, protein kinase C) to alter cellular processes [25].

Corticosteroids may cause rapid effects by changing the physicochemical properties of the cell membrane. The lipophilic corticosteroid molecules intercalate into the phospholipid bilayers of cellular membranes. Corticosteroids acutely inhibit extraneuronal uptake of norepinephrine in the smooth muscle cells isolated from human bronchial arteries [26] which will probably increase the duration of the norepinephrine/vasoconstrictor signal, and consequently decreases airway blood flow as seen in healthy and asthmatic subjects after inhalation of corticosteroids [27].

ICS have been shown to acutely suppress airway hyperperfusion associated with asthma. A single dose of inhaled fluticasone propionate has been shown to decrease airway mucosal blood flow in healthy and asthmatic subjects with a maximal effect, 30 min after inhalation, and a return to baseline at 90 min [27]. The blood flow effect increased in a dose-dependent manner up to 880 mg of fluticasone propionate, with a significantly greater effect in asthmatics than in healthy controls. The acute vasoconstrictor action has also been demonstrated after inhalation of beclomethasone dipropionate and budesonide [28].

Moreover, ICS has been shown to suppress the increased microvascular permeability and plasma leakage into the airway lumen, which adds to the airway obstruction in asthma, as determined by measuring concentrations of high molecular weight proteins (e.g. alpha-2-macroglobulin) in induced sputum [29, 30] and bronchoalveolar lavage fluid [31, 32].

Based on interventional studies, the inhaled daily doses and the length of therapy seem to be the critical determinants of the vascular effects of ICS. Furthermore, the inhibitory effects on the remodeling process seem to occur only with long-term therapy with corticosteroids. Whereas a 6-month treatment with a daily dose of 800 mg beclomethasone dipropionate reduced the number of blood vessels and the vascular area [33], a 6-week treatment with fluticasone propionate was only effective at a daily inhaled dose of 1000 mg, and not at 200 mg, to reduce significantly the number of blood vessels and the vascular area [34].

### Functional effects of inhaled corticosteroids

ICS prevents the late but not the early allergic response. However, prolonged treatment with ICS is found to be effective in reducing the early response to an allergen challenge in a time-dependent and probably dose-dependent way [35, 36]. Moreover, ICS do not protect against bronchoconstriction when given immediately before exercise [37]. Regular treatment with ICS is effective in reducing bronchial responsiveness to direct and indirect stimuli [38] and reduces the prevalence and the severity of exercise-induced asthma [37].

### Interaction of inhaled corticosteroids with some asthma medications

Corticosteroids increase the expression of β2-adrenergic receptors in the lung and prevent their down regulation and uncoupling in response to β2-agonists [39]. The corticosteroid-induced decrease in airway blood flow is likely to enhance the action of inhaled bronchodilators by diminishing their clearance from the airway. Since the corticosteroid-induced vasoconstriction peaks rapidly (~30 min after drug inhalation), simultaneous administration of ICS and bronchodilators is likely to be of clinical significance [11].

β2-agonists enhance the action of corticosteroids, with an increase in nuclear translocation of GRs in vitro [40] and enhanced suppression of inflammatory genes [41]. Nuclear localization of GRs is also enhanced after treatment of asthmatic patients with a combination inhaler compared with the same dose of inhaled steroid given alone [42].

Low doses of theophylline significantly increase histone deacetylase (HDAC) activity in bronchial biopsy specimens from asthmatic patients, and the increase in HDAC activity is correlated with the reduction in airway eosinophils. Because corticosteroids also activate HDAC, but via a different mechanism, theophylline should enhance the anti-inflammatory actions of corticosteroids; this enhancement occurs because the HDAC recruited to the inflammatory gene will be more effective at switching off the gene. Indeed, therapeutic concentrations of theophylline markedly potentiate the anti-inflammatory effects of corticosteroids in vitro [43]. This effect may explain why adding a low dose of theophylline is more effective than increasing the dose of ICS in patients whose asthma is not adequately controlled [44, 45].

### Inhaled corticosteroid resistance

Relative resistance is seen in patients who require high doses of inhaled and oral steroids (steroid-dependent asthma). Biopsy studies have demonstrated the typical eosinophilic inflammation of asthma in those patients [46].

Persistent immune activation and airway inflammation, which to varying degrees is resistant to glucocorticoid therapy, appears to define the immunologic abnormality underlying steroid-resistant asthma [47]. Certain cytokines (particularly interleukin-2, interleukin-4, and interleukin-13, which show increased expression in bronchial biopsy samples from patients with steroid-resistant asthma) may induce a reduction in affinity of GRs in inflammatory cells, such as T-lymphocytes, resulting in local resistance to the anti-inflammatory actions of corticosteroids [46, 48]. Moreover, the inhibitory effect of corticosteroids on cytokine release is reduced in peripheral blood mononuclear cells from patients with steroid-resistant and steroid-dependent asthma [49].

Other mechanisms involved include impaired nuclear localization of GRs in response to a high concentration of corticosteroids and defective acetylation of histone-4, interfering with the anti-inflammatory actions of corticosteroids [50].

## Dosage and therapeutic regimens

Inhaled corticosteroids are the mainstay of pharmacotherapy for asthma control. Multiple national and international guidelines on asthma have been published [2, 3, 51–60] (Table 2), each with varying recommendations and level of detail regarding the use and rationale of ICS. In some, recommendations on the dose of ICS for initiation and step-up therapy are age-group based. What is common to these guidelines are:

**Table 2:** Overview of national and international guidelines on asthma in children

Guideline	Last updated	Age categories (years)	Preferred step up choices after initial low dose ICS	Specifies indications for starting low dose ICS	Specifies indications for starting moderate dose ICS	Specifies indications for starting high dose ICS	Describes adverse effects	Review interval for dose drop
GINA	2016	Up to 5	Mod dose ICS or add LTRA	Yes	Yes	Yes	Yes	3 months
		6–11	Mod dose ICS					
		12 and older	Add LABA					
Australia	2015	0–1	NS	Yes	NS	NS	NS	NS
		1–2	NS					
		2–5	NS					
		6 and older	NS					
Canada Preschool	2015	1–5	Mod dose ICS	Yes	Yes	NS	NS	3 months
Canada Older	2012	6–11	Mod dose ICS	Yes	NS	NS	Yes (CTS 2010)	weeks to months
		12 and older	Add LABA					
SIGN	2014	0–4	LTRA	Yes	NS	NS	Yes	3 months
		5–12	Add LABA					
		13 and older	Add LABA					
ICON	2012	Describes other guidelines	Describes other guidelines	NS	NS	NS	NS	NS
Saudi Arabia (SINA)	2012	<5	Mod dose ICS	Yes	NS	NS	Yes	3–6 months
		5 and older	Add LABA					
Japan	2012	<2	LTRA is first choice, not ICS. Step-up: mod dose ICS	Yes	Yes	Yes	NS	3 months
		2–5	Mod dose ICS					
		6–15	LABA or mod dose ICS					
South Africa	2009	<5	Mod dose ICS	Yes	NS	NS	Yes	3 months
		5 and older	LTRA or LABA or mod dose ICS					
PRACTALL	2008	0–2		NS	NS	NS	Yes	NS
		3–5	Mod dose ICS or add LTRA					
		6 and older	Mod dose ICS or add LTRA					
NHLBI	2007	0–4	Mod dose ICS	Yes	NS	NS	Yes	3 months
		5–11	Mod dose ICS or add LABA					
		12 and older	Add LABA					
India	2003	<5	Mod dose ICS	Yes	NS	NS	Yes	3–6 months
		5 and older	Add LABA

- The need for step-up therapy based on poor asthma control
- Recognition that preschoolers are different from older children in terms of diagnostic and treatment procedures
- A recommended step-down interval of three months of good asthma control
- The consideration of ICS dose pertaining to growth.

### ICS regimes

Perhaps the best-known guideline is that of the Global Initiative for Asthma (GINA), which was last updated in April 2015. As one of the most recent and comprehensive guidelines, it details [3]

- The range of low, moderate and high doses of ICS for children aged up to 5 years, 6–11 years, and 12 years or older separately (Table 3)

**Table 3:** ICS doses by formulation and age

Drug	Daily dose ug (age ≤ 5 years)	Daily dose ug (age 6–11 year)			Daily dose ug (age ≥ 12 years)		
	Low^a^	Low	Medium	High	Low	Medium	High
Betamethasone Dipropionate (CFC)	-	100–200	>200–400	>400	200–500	>500–1000	>1000
Betamethasone Dipropionate (HFA)	100	50–100	>100–200	>200	100–200	>200–400	>400
Budesonide (pMDI + spacer)	200	-	-	-	-	-	-
Budesonide (DPI)	-	100–200	>200–400	>400	200–400	>400–800	>800
Ciclesonide	160	80	>80–160	>160	80–160	>160–320	>320
Fluticasone propionate (DPI)	-	100–200	>200–400	>400	100–250	>250–500	>500
Fluticasone propionate (HFA)	100	100–200	>200–500	>500	100–250	>250–500	>500
Mometasone furoate	Not studied below 4 years	110	220–<440	≥440	110–220	>220–440	>400
Triamcinolone acetonide	Not studied	400–800	>800–1200	>1200	400–1000	>1000–2000	>2000

*HFA* hydrofluoroalkane propellant

^a^A low daily dose is defined as the dose that has not been associated with clinical adverse effects in trials that included measures of safety

Modified from GINA 2015 [3]
- The indications to start ICS at low, moderate and high doses (Tables 4 and 5) which is also mirrored in the Japanese guidelines [51]

**Table 4:** Indications for initial controller therapy in children aged 6 years and above

Presenting symptoms	Preferred initial controller (Strength of evidence)
Infrequent asthma symptoms, but has one or more risk factors for exacerbations (see below)	Low dose ICS (Evidence D)
Asthma symptoms or need for SABA between twice a month and twice a week; or patient wakes due to asthma one or more times a month	Low dose ICS (Evidence B)
Asthma symptoms or need for SABA more than twice a week	Low dose ICS (Evidence A)
Troublesome asthma symptoms most days; or waking due to asthma once a week or more, especially if any risk factors exist (see below)	Moderate/high dose ICS (Evidence A), or Low dose ICS/LABA (Evidence A)#
Initial asthma presentation is with severely uncontrolled asthma, or with an acute exacerbation	Short course of oral corticosteroids AND start regular controller treatment: - High dose ICS (Evidence A), or - Moderate dose ICS/LABA (Evidence D)#
Risk Factors for Exacerbations: Uncontrolled asthma symptoms High SABA use (with increased mortality if > one 200-dose canister per month) Inadequate ICS (not prescribed ICS; poor adherence; incorrect inhaler technique) Low FEV_1_, especially if <60 % predicted Major psychological or socioeconomic problems Exposures: Smoking; allergen exposure if sensitized Comorbidities: obesity; rhinosinusitis; confirmed food allergy Sputum or blood eosinophilia Pregnancy Ever intubated on in intensive care for asthma At least one severe exacerbation in the last 12 months	
Risk factors for developing fixed airflow limitation: Lack of ICS treatment Exposures: tobacco smoke; noxious chemicals; occupational exposures Low FEV_1_; chronic mucus hypersecretion; sputum or blood eosinophilia	

Legend:

*LABA* long acting beta2-agonist, *SABA* short acting beta_2_-agonist

# = not recommended in children aged 6–11 years

Evidence A – data from randomized controlled trials and meta-analyses, rich body of data

Evidence B - data from randomized controlled trials and meta-analyses, limited data

Evidence C – data from nonrandomized trials/observational studies

Evidence D – panel consensus judgment

Modified from GINA 2015 [3]

**Table 5:** Indications for initial low-dose ICS controller therapy in children aged 5 years and below

Indication to start low-dose ICS: Symptom pattern consistent with asthma (Box 1) and asthma symptoms not well-controlled (Box 2), or at least 3 exacerbations per year OR Symptom pattern not consistent with asthma, but wheezing episodes occur frequently (e.g. every 6–8 weekly)			
BOX 1: Features suggesting a diagnosis of asthma in children 5 years and younger			
Feature	Characteristics suggesting asthma		
Cough	Recurrent or persistent non-productive cough that may be worse at night or accompanied by some wheezing and breathing difficulties Cough occurring with exercise, laughing, crying or exposure to tobacco smoke in the absence of an apparent respiratory infection		
Wheezing	Recurrent wheezing, including sleep or with triggers such as activity, laughing, crying or exposure to tobacco smoke or air pollution		
Difficult or heavy breathing or shortness of breath	Occurring with exercise, laughing or crying		
Reduced activity	Not running, playing or laughing at the same intensity as other children; tires earlier during walks (wants to be carried)		
Past or family history	Other allergic disease (atopic dermatitis or allergic rhinitis) Asthma in first-degree relatives		
Therapeutic trial with low dose ICS and as-needed SABA	Clinical improvement during 2–3 months of controller treatment and worsening when treatment is stopped		
BOX 2: GINA assessment of asthma control in children 5 years and younger			
Symptoms in the last 4 weeks	Level of control		
	Well controlled	Partly controlled	Uncontrolled
Daytime asthma symptoms for more than a few minutes, more than once a week	None	1–2 of these	3–4 of these
Any activity limitation due to asthma (Runs/plays less than other children, tires easily when walking/playing)			
Reliever medication (excludes reliever taken before exercise) needed more than once a week			
Any night waking or night coughing due to asthma			

Legend:

*SABA* short acting beta_2_-agonist

Evidence A – data from randomized controlled trials and meta-analyses, rich body of data

Evidence B – data from randomized controlled trials and meta-analyses, limited data

Evidence C – data from nonrandomized trials/observational studies

Evidence D – panel consensus judgment

modified from GINA 2015 [3]
- The evidence for use of ICS in an acute asthma exacerbation (mirrored in the guidelines of the Canadian Thoracic Society [52, 53] and Scottish Intercollegiate Guidelines Network (SIGN) [54].

Of all the guidelines, only that of the SIGN [54] mentions a recommended ICS dosing frequency of twice-daily rather than once-daily, for reasons of superior efficacy. The other guidelines make no recommendations regarding dosing frequency.

In general, the consensus is the need to establish the diagnosis of asthma. In cases where this is unclear (such as in preschool children) and other known causes such as chronic infection, a therapeutic 2–3 month-long trial of low-dose ICS appropriate to age and formulation type can be initiated to assess the response of symptoms to this treatment.

Low-dose daily ICS is the first-line controller therapy for mild persistent asthma. None of the guidelines espouse intermittent ICS as an option; this is borne out by a recent meta-analysis [61] in children aged 1 year and older with suspected persistent asthma. The authors found similar rates in use of rescue oral corticosteroids; however, daily ICS was superior to intermittent ICS in several parameters of lung function, airway inflammation, asthma control, and reliever use.

The TREXA trial [62] demonstrated both the superiority of daily ICS over intermittent ICS. Of note was the suggestion that intermittent ICS was superior to no ICS in children with asthma that had been controlled through recent ICS use, though this second finding did not reach statistical significance (hazard ratio 0.62, 95 % CI 0.37–1.05, *p* = 0.073). These results arose from the 4-week run in period to establish asthma control before the participants were randomized to the 4 arms that led to the abovementioned results.

Recent guidelines advocate that when low-dose daily ICS is insufficient, increasing the dose of ICS is the preferred step-up therapy compared to add-on therapy with other agents in children less than 12 years of age. Where the child is 12 years and older, addition of a long-acting beta-agonist (LABA) to the existing ICS dose is preferred.

### Small-particle ICS in children

Amirav et al [63] has described the differences in the airways and air flow between infants, older children, and adults. The importance of mass median aerodynamic diameter of delivered aerosol particles to children – specifically, small particles may improve lung deposition and thus, efficacy – was described theoretically as well as summarized from existing in-vitro and in-vivo studies. The limitation of these studies was the absence of an in-vivo, comparative study on the efficacy of small-particle ICS on asthma control. van Aalderen et al [64] recently demonstrated better asthma control and lower severe exacerbation rates in a matched retrospective cohort analyses of children aged 5–11 years, when using small-particle beclomethasone dipropionate hydrofluoroalkane compared to fluticasone propionate, in both initiation as well as step-up therapy. When a higher dose of small-particle beclomethasone was compared to addition of LABA as step-up therapy for asthma control, outcomes were generally similar, though the use of short-acting beta-agonists (SABA) was lower in the small-particle beclomethasone group. Among the limitations of this study was the lack of information on side effects, such as growth, which would be a potential concern in the cohort using higher doses of ICS.

### Role of long acting ẞ2 agonists and leukotriene receptor antagonists in initiation of controller therapy

In a meta-analysis that compared initiation of LABA/ICS, versus ICS of the same dose in steroid-naïve adults and children aged 6 years and above for asthma control, the LABA/ICS group had slight reduction in symptoms and rescue β2-agonist use but there was no difference to exacerbations requiring oral corticosteroids or rate of hospital admissions [65].

Various clinical trials [66–71] have demonstrated that ICS is superior to LTRA as monotherapy for asthma control; most of these studies were in children aged 6 years and older, with the exception of Szefler et al. [67] who demonstrated the superiority of ICS over LTRA in children aged 2–8 years. In contrast, in a large retrospective cohort study in children aged 4–17 years, LTRA appeared to be as effective as ICS upon initiation for asthma control. The authors acknowledged that this result could have been confounded by the ICS group having had more severe asthma at baseline [71].

The use of Budesonide/Formoterol as a single-inhaler therapy (SiT; also known as Symbicort Maintenance and Reliever Therapy SMART) for asthma control as well as as-needed relief is licensed and has been advocated in patients 12 years and above. A Cochrane meta-analysis [72] concluded that SiT was superior to fixed-dose combination inhalers in terms of reducing the number of exacerbations requiring oral steroids, hospitalizations and emergency room visits, whilst using a lower mean daily dose of ICS. In terms of reducing exacerbations, SiT proved superior to the other treatment groups: fixed-dose budesonide/formoterol with as-needed terbutaline, and four-times-as-high daily ICS dose (320micrograms of budesonide) with as-needed terbutaline. In addition, growth was superior in the SiT group compared to the higher-dose budesonide group [73, 74].

In summary, for initiation, ICS is preferable to LTRA as monotherapy, with no significant difference between LABA/ICS versus ICS of the same dose. Among the options for LABA/ICS, SiT seems promising, but more studies need to be conducted in children less than 12 years of age.

### Step-up controller therapy

In a meta-analysis that compared the addition of LABA to ICS versus ICS of the same dose in adults and children aged 4 years and above with persistent asthma, revealed that LABA/ICS was superior to same-dose ICS alone in markers of lung function. For the whole group, LABA/ICS was also superior to a higher ICS dose in terms of peak expiratory flow and growth rate. However, in children aged 4–18 years (mean 10 years), there was a higher risk of hospitalization and of exacerbations requiring oral steroids in the LABA/ICS compared to higher ICS dose, but this did not reach statistical significance; the authors urged that further studies in this area are needed [75]. A more recent clinical trial showed no difference between salmeterol-fluticasone compared to a double dose of fluticasone in children aged 6–16 years with symptomatic asthma despite the double-dose group receiving 200 micrograms twice daily of fluticasone propionate [76].

Another meta-analysis that compared addition of LTRA to ICS, compared to ICS of the same or higher dose, was limited by the few trials available, with data available only in children aged 6 years and above [77]. Three trials compared LTRA + ICS to ICS of the same dose, with no significant clinical benefit of the addition of LTRA. Only one triple-crossover trial in children aged 6–17 years compared LTRA + ICS to a higher dose of ICS, again with no difference in hospitalizations or need for oral steroids observed between the two groups. Most significant in that same study was the finding that LABA + ICS was superior to LTRA + ICS; the authors used a composite endpoint comprising less need for oral prednisone, better asthma control or superior FEV1 but did not show the breakdown of results for each separate endpoint. This study also highlighted the importance of follow-up and customizing pharmacotherapy, as there may be a differential response in children to different options of step-up therapy [78].

A third meta-analysis that compared LABA to LTRA as adjunctive therapy supports the use of LABA/ICS over LTRA as an adjunct, as evidenced by reduction of exacerbations requiring oral corticosteroids, rescue therapy use, and symptoms; and superior lung function and quality of life. However, most of the trials included were in adults and adolescents; there was a lack of data in children aged 5 years and below [79].

In summary, LABA/ICS appears superior to ICS or LTRA as step-up therapy. However, this requires further study, especially in preschoolers.

### Role of ICS in acute asthma exacerbations

The GINA [3] and Canadian [52, 53] guidelines discuss the benefit of ICS during an acute asthma exacerbation. Current evidence in children does not support dose escalation of ICS in patient self-management of an acute asthma exacerbation. Studies in adults had suggested early dose escalation of existing ICS to double, or even four to five times the original, may be of benefit during an asthma exacerbation [80, 81], but did not significantly reduce the need for oral corticosteroids [82]. Studies in children did not demonstrate any reduction in hospitalization or use of oral corticosteroids, though there was suggestion of symptomatic improvement with higher doses of ICS [83–86]. A recent Cochrane review [87] did not find a significant reduction in the need for oral corticosteroids in older school-aged children, which were drawn from the TREXA study; [62] intermittent ICS did show symptomatic improvement and lower likelihood of requiring oral corticosteroids in preschoolers, but the authors acknowledged that this was a distinct group that might not necessarily have long-term asthma. Given the lack of safety information of very high doses of ICS in children, especially if administered frequently, we do not recommend that children be given high doses of ICS to try to mitigate an acute asthma exacerbation.

The role for ICS in the setting of asthma exacerbations presenting to the emergency department, is more promising. Edmonds et al [88] that showed that ICS reduced hospitalizations in acute asthma where patients did not receive systemic steroids. The addition of ICS to standard asthma treatment that included systemic corticosteroids, also helped prevent hospitalizations. There were no significant adverse effects from the use of ICS in this situation. However, there was wide variation in the type and doses of ICS used, with the most frequent ICS used being Budesonide at a median dose of 900 micrograms each, and a median cumulative dose of 2000 micrograms over up to 6 h. Two, more recent meta-analyses that compared ICS to systemic corticosteroids in the emergency department for children presenting with acute asthma exacerbations, found no difference between ICS and systemic corticosteroids in hospitalization rates [89, 90]. Beckhaus et al [89] also found no difference between ICS and systemic corticosteroids in the outcomes of unscheduled visits for asthma symptoms (relative risk 9.55; 95 % CI: 0.53–170.52), or need for additional courses of systemic corticosteroids (relative risk 1.45; 95 % CI: 0.28–7.62). However, there was significant heterogeneity between the primary studies including route, type and dose of ICS; and these additional results had extremely wide confidence intervals.

In conclusion, there is insufficient evidence to recommend that ICS can replace systemic corticosteroids in the emergency department attendance for a child with an acute asthma exacerbation.

### Future needs in research

Small-particle ICS and SiT represent two promising approaches that warrant further study. Small-particle ICS appears superior to LABA/ICS in both initiation and step-up therapy, but this inference is restricted to a single retrospective cohort analysis, and a single comparator (fluticasone propionate); prospective randomized controlled studies are required. SiT may be ideal when considering LABA/ICS, both in terms of evidence as well as convenience, but it is licensed and studied mainly in children 12 years and older; promising results in younger children should be substantiated with further study.

## Methods of ICS delivery

Inhaled medications are only effective if they are used properly. The correct use of an inhaler device delivers the medication right to the lungs insuring a better response. The proper inhalation technique is not followed by most of asthma individuals. By training and practice, one can learn and adhere to proper inhalation techniques and practice.

### Choosing the appropriate device

Currently available devices for delivering inhaled medications include jet nebulizers, ultrasonic nebulizers, metered dose inhalers (MDIs) with and without a spacer device, and dry powder inhalers (DPIs). An important factor is the cooperation between the child and caregiver. Jet nebulizers may be more easy to use in infants and toddlers as they need no active cooperation. With the improvement of cooperation throughout childhood, MDIs with holding chambers and masks should be used. Finally, MDIs with holding chambers or DPIs can be used in fully cooperative older children and adolescents.

To date, there is no ideal device for delivering inhaled medications. Characteristics for the perfect device include reproducible dose delivery to the lungs across a wide range of inspiratory flows; this is important due to the uneven inspiratory rate by children. The optimum size of the particles (2–5 microns) is necessary to optimize drug delivery. Particles larger than 10 microns deposit in the oropharynx, particles 5–10 microns deposit in the trachea and large bronchi, and particles less than one micron are exhaled. Other desirable characteristics include ease of use, portability, and the availability of dose counters. There is capacity of dispensing single dose by DPIs, but multiple dose capacity devices are also used [54, 59].

Infants and young children (below 5 years of age) represent a unique subpopulation with significant difficulties and challenges for aerosol delivery due to peculiar anatomic, physiological, and emotional factors. Infants are obligate nose breathers and this may lead to less effective lung deposition of aerosols and particles. They also fail to hold their breath leading to exhalation of a great proportion of the inhaled medication. Crying is another important factor that may cause deposition of the drug in the upper respiratory tract and affect the seal between the mask and face [91]. Even a 1-cm gap between the mask and the face may reduce the dose delivered to the respiratory tract by 50 % [92] It was suggested that large-particle corticosteroid aerosols are not likely to be effective in infants and young children [91] and that small particles may offer better lung deposition [63, 64].

### Data comparing different inhalation devices

A systematic review was conducted to determine the clinical effectiveness of pressurized MDIs with or without spacer compared with other hand held inhaler devices for the delivery of corticosteroids in stable asthma. Twenty four randomized controlled trials were included. There was no evidence pertaining to the effectiveness of alternative inhaler devices (DPIs, breath actuated pressurized MDIs, or hydrofluoroalkane pressurized MDIs) when compared to the pressurized MDIs for delivery of inhaled corticosteroids. Hence, in the first line delivery devices pressurized MDIs remain the most cost effective [93]. A Cochrane systematic review assessed the efficacy and safety of inhaled corticosteroids delivered via nebulizer versus holding chamber for the treatment of chronic asthma. The only double-blinded study included revealed that high dose of budesonide delivered by the particular nebulizer was more effective than budesonide 1600 ug delivered via a large volume spacer [94].

There was equal efficacy, acceptable safety and tolerability profile when beclomethasone dipropionate (BDP) suspension for nebulization 3000–4000 μg day-1 given via a nebulizer was compared with BDP spray 1500–2000 μg day-1 given via a metered dose inhaler in steroid-dependent adult patients with moderate to severe asthma [95]. The results from another study attested to the efficiency of jet-nebulized budesonide suspension and indicated that nebulized budesonide is equipotent to standard budesonide therapy delivered by pressurized metered dose inhaler, provided nebulization is synchronized with inspiration and no loss of aerosol occurs during expiration [96]. On the other hand, a randomized placebo-controlled study compared the relative lung delivery of fluticasone propionate (FP), using adrenal suppression as a surrogate for the respirable dose, when administered via large volume spacer or nebulizer in healthy adults. The spacer produced about a sevenfold higher relative lung dose than the nebulizer. The authors suggested that very little of the labeled nebulized dose is actually respirable [97]. In a double-blind cross-over study, 40 children with childhood asthma were randomized to receive 400 micrograms BDP as aerosol or powder. The peak flow performance in the morning was more benefited in the aerosol group with severe asthma. However there was no difference in evening peak flow and symptom scores [98].

In the short-term treatment of mild to moderate asthma in children, BDP delivered by Clickhaler was a well-tolerated and therapeutically equivalent alternative to BDP delivered by a conventional MDI plus spacer [99]. A randomized cross over study showed that in 3- to 4-year-old children, budesonide dose delivery was higher and/or more consistent from the pMDI Nebuhaler than from the Turbuhaler [100]. One hundred thirty seven patients received FP 250 μg twice daily from a Diskus inhaler and 140 received budesonide 600 μg twice daily from a Turbuhaler inhaler for 4 weeks. The Diskus inhaler was generally rated more highly by patients than the Turbuhaler [101].

The electrostatic and non electrostatic properties of metal inhalers also regulate the amount of inhalation. There is greater delivery of aerosol to the mouth by the metal valved holding chamber (VHC) having non electrostatic properties compared to the plastic polypropylene VHC. In young children, when budesonide was used (400 ug/day) under real life conditions, the metal VHC was not associated with greater hypothalamus pituitary axis (HPA) suppression [102]. The metal VHC was not more effective than the Aero Chamber despite a greater total dose delivered to the mouth [103].

The type of spacer did not influence the state of asthma control in 141 patients 5–57 months old but the parents’ preference made a difference in choice [104]. There was improved perception by parents of their children’s asthma control by better Pediatric Asthma Caregiver’s Quality of Life (PACQLQ) scores when the valved holding chamber has been enhanced to include a Flow-Vu inspiratory flow indicator that provides visual inhalation feedback during use although there was no actual change in asthma control [105]. A recent trial demonstrated that Salmeterol/fluticasone combination given through the breath actuated inhaler (BAI) produces comparable efficacy and safety endpoints as metered dose inhaler. Additionally, BAI was more preferred by patients compared to conventional MDI [106]. This shows that there is individual preference in choosing the inhaler device that could be an influential factor in decision making by the health care professional.

In summary, age and cooperation between the child and caregiver are important determinants of effective inhalation therapy. Other important factors include the particle size, device portability, and the availability of dose counters. Due to the relatively flat dose–response relationship in asthma, significant benefit in terms of symptom and lung function improvement is usually seen at low to moderate doses of different ICS.

## Adverse effects of ICS

Adverse effects resulting from the use of ICS are often underestimated in daily clinical practice. Although ICS treatment is generally considered safe in children, the potential adverse effects related to its regular use have been and continue to be a matter of concern. ICS are reported to cause some local and systemic adverse effects [107] (Table 6). This seems to be dose-dependent being most common in individuals receiving high dose ICS with or without oral corticosteroids (OCS).

**Table 6:** Potential local and systemic side effects of inhaled corticosteroids

Local	Systemic
Pharyngitis	Suppressed HPA-axis function
Dysphonia	Adrenal crisis (with insufficiency)
Reflex cough	Suppressed growth velocity
Bronchospasm	Decreased lower-leg length
Oropharyngeal candidiasis	Reduced bone mineral density
	Suppressed HPA-axis function
	Bone fractures
	Osteoporosis
	Skin thinning
	Skin bruising
	Cataracts
	Glaucoma

Modified from Dahl R [107]

*HPA-axis* hypothalamic pituitary adrenal axis

### Local adverse effects

Relatively few studies sought to evaluate local side effects of ICS as they are generally viewed as minor complications of therapy. Nevertheless, approximately 5–10 % of subjects treated with ICSs report adverse effects in the oral cavity. The local effects can be clinically significant, affect patient quality of life, and hinder compliance with therapy [108–110]. Local deposition of glucocorticoids is, thus, an important risk factor for oropharyngeal candidiasis [111, 112].

A meta-analysis revealed that the ICS MDI device was associated with a 5-fold greater risk of oral candidiasis versus MDI placebo (OR, 5.40). In contrast, the ICS DPI device had a 3-fold greater risk for oral candidiasis versus DPI placebo (OR, 3.24). A similar trend was observed with regard to dysphonia. Both ICS MDI and DPI were associated with an approximately 2-fold greater risk of pharyngitis compared with placebo [113].

#### Oral candidiasis

Oropharyngeal candidiasis clinically presents as white soft plaques that leave a painful erythematous, eroded, or ulcerated surface. Common sites are the buccal mucosa, oropharynx, and lateral aspects of tongue. Patients may complain of tenderness, burning, and dysphagia once the pseudo-membrane gets disrupted [112, 114]. The mechanism of oral candidiasis induced by ICS remains obscure. Predictive factors identified by multivariate logistic regression were higher daily dose of ICS and concomitant use of OCS [115].

Inhaled fluticasone use and dysphonia were reported in association with posterior pharyngeal candidiasis at bronchoscopy even in the absence of clinically overt oral involvement [116]. Isolated Candida was significantly greater in patients with oral symptoms than asymptomatic patients [117].

Esophageal candidiasis was reported with the use of dry powder budesonide which might have favored esophageal drug deposition [118]. This occurred in an 18-month old girl who received ICS therapy for bronchial asthma who presented with coffee-ground emesis and melena. The diagnosis was based on the presence of pseudohyphae in endoscopic biopsy and the identification of candida species by culture [119]. The prevalence of esophageal candidiasis was 37 % among a group of Japanese patients treated with inhaled fluticasone propionate, compared to 0.3 % of the control patients. A reduction in the daily dose eliminated the infection [120]. Nevertheless; another investigation revealed that subclinical esophageal and oropharyngeal Candida colonization was statistically comparable between asthma patients on ICS and steroid naive asthmatic controls suggesting that the risk of esophageal candidiasis due to ICS is low [121].

#### Dysphonia

Rachelefsky et al. [113] analyzed data from 23 studies published from 1966 through 2004 and noted that ICS at all dosages was associated with a 5.2-fold greater risk of dysphonia as compared with placebo. Causes of dysphonia associated with ICS therapy have been poorly investigated, and the origins of dysphonia may have multiple confounding factors. It may likely result from deposition of active ICS in the oropharynx during inhalation resulting in chemical inflammation [122, 123]. Candidiasis of the larynx was infrequently observed in patients with voice complaints after ICS therapy [124].

Using the lowest effective dosage of ICS and administering it with a spacer may decrease oropharyngeal deposition of inhaled aerosols. After using a spacer, it must be washed with tap water and allowed to air dry. Patients should be instructed to rinse the mouth, gargle, and wash the face after inhalation [125]. Switching to another ICS was also suggested [126].

#### Other local complications

Xerostomia may associate inhalation therapy and is clinically presented as oral fissuring, ulceration, and epithelial atrophy. As a consequence of diminished salivary flow, food retention and an acidic environment is encouraged. This in turn encourages the growth of aciduric bacteria and dental caries [112]. Caries was detected in mixed or permanent dentition of children receiving various forms of inhalation therapy [125, 126]. However, the causal relationship to ICS in particular and long-term effects have yet to be established.

Inhaled drugs in general can also alter the taste perception due to interaction of drug metabolite and saliva, or secondary to xerostomia and/or candidiasis [112]. Halitosis and gingivitis could also happen due to oral infections and xerostomia [127, 128]. Mouth breathing habit in these patients further increases gingivitis due to dehydration of the alveolar mucosa [112].

Immediate mouth washing after inhalation was found to be most useful for prevention of oral complications and the amount of drugs removed by mouth washing is significantly associated with the time lag between inhalation and mouth washing [129].

### Systemic adverse effects

#### Growth delay

Asthma as a chronic disease by itself has growth-suppressing effects probably due to growth-suppressing influence of endogenous cytokines and glucocorticoids produced in response to illness and inflammation and this can confound studies of the effect of ICS on growth. Any resulting delay in the growth process is associated with delays in pubertal development and pronounced growth deceleration in late childhood [130]. Normal childhood growth can be divided conceptually into three phases according to primary growth-supporting factors: nutrition-dependent growth of infancy, growth hormone (GH)-dependent childhood growth, and sex steroid/GH stimulation of pubertal growth. Susceptibility to glucocorticoid-induced growth suppression appears to increase during periods of transition from one phase to another, particularly in the immediate prepubertal years [131]. The effect of ICS on growth velocity and final adult height has been a subject of debate.

Table 7 displays some studies that sought to investigate this adverse effect [132–141]. Growth suppression was considered both a sensitive and relatively specific indicator of systemic corticosteroid effect. Regular use of ICS at low or medium daily doses was associated with a mean reduction of 0.48 cm/year in linear growth velocity and a 0.61-cm change from baseline in height during a one-year treatment period in children with mild to moderate persistent asthma [142]. An evidence-based analysis revealed that there is ICS dose-dependent reduction in growth velocity in prepubescent school-aged children with mild to moderate persistent asthma whatever the ICS molecule (mometasone, ciclesonide or fluticasone) consumed [143]. Flunisolide hydrofluoroalkane has not been associated with reduced growth velocity in a 12 month study [138]. In a randomized controlled trial on 4–9 year old children, one year of treatment with a daily dose of 200 μg of mometasone furoate DPI in the morning resulted in some changes in growth velocity when compared with placebo [139].

**Table 7:** Summary of some studies on the effect of inhaled corticosteroids on linear growth

Authors	Subjects number (age)	Study duration	Study type	ICS used	Control treatment	Outcome
van Bever et al. [132]	42 (5–18 years)	Variable	Retrospective	Beclomethasone dipropionate	NA	Final adult Ht comparable between ICS and control groups. However, difference between adult Ht and target Ht greater in ICS group.
Inoue et al. [133]	61 (6–17 years)	Adult Ht recorded at 20 year	Retrospective	Beclomethasone dipropionate 300–800 ug/day	NA	Unaffected mean difference in adult Ht between ICS and control group (0.14, 95 % CI-0.38–0.65).
Skoner et al. [134]	670 (0.5–8 years)	One yr	DBRCT	Budesonide 500–1000 ug/day	Theophylline; Beta-2 agonist; Cromolyn	Growth reduction 0.84 cm (95 % CI 1.51–0.17) in ICS group than placebo
Agertoft and Pedersen [135]	142 (3–14 years)	13 years	Prospective	Budenoside 110–877 ug/day	NA	Unaffected adult Ht (mean final minus predicted 0.31 cm (95 % CI 0.6–1.2).
Pauwels et al. [136]	7241 (5–66 years)	Three yr	Prospective DBRCT	Budesonide 400 μg/day for adults and 200 ug for children < 11 year	Placebo	In children < 11 years, growth was reduced in the ICS group by 1.34 cm. The reduction was greatest in the first year of treatment (0.58 cm) than years 2 and 3 (0.43 cm and 0.33 cm, respectively).
Stefanovic et al. [137]	28 (1.5–4.3 years)	One yr	Prospective (no control group)	Fluticasone propionate 100–200 ug/day	NA	No effect on linear growth or on the HPA-axis.
Bensch et al. [138]	218 (4–10 year)	One yr	Prospective DBRCT	Flunisolide 340 μg/day	Placebo	Mean growth velocity [6.01 ± 1.84 cm/52 weeks] for ICS was comparable to placebo [6.19 ± 1.30 cm/52 weeks] (*P* = 0.425).
Skoner et al. [139]	187 (4–9 years)	1.25 years	Prospective RCT	Mometasone furoate 100 or 200 ug/day	Placebo	A total daily dose of 100 μg had no effect, whereas 200 μg led to some changes in growth velocity as compared to placebo.
Kelly et al. [140]	943 from CAMP Study	4–6 years. Adult Ht recorded at 24.9 ± 2.7 years	Prospective RCT	Budesonide 400 μg/day	Nedocromil or placebo	1.2 cm reduction of adult Ht in the ICS group compared to placebo. A larger daily dose in the first 2 years led to a lower adult Ht (-0.1 cm for each ug per Kg body Wt.
Protudjer et al. [141]	2746 from a population-based birth cohort	12 years. Ht recorded at 8 and 12 years	Prospective	Variable	Variable	Asthmatics using ICS were 1.28 (95 % CI 0.62–1.95) shorter than those not using ICS. No consistent association between asthma and pubertal staging.

*ICS* inhaled corticosteroids, *DBRCT* double blind randomized controlled trial, *RCT* randomized controlled trial, *NA* data not available

The effect of childhood ICS use on final adult height is conflicting [129]. The Childhood Asthma Management Program (CAMP) trial is the only prospective study that was started at childhood and followed the subjects to adulthood. Although the attained height was not a primary objective of this trial, the height was monitored regularly, frequently, and precisely with a stadiometer [144]. A larger daily dose of inhaled glucocorticoid in the first 2 years was associated with a lower adult height (−0.1 cm for each microgram per kilogram of body weight; *p* = 0.007) [140]. Loke et al. [145] recently noted a slight reduction of about one cm in final adult height which when interpreted in the context of average adult height in England represented a 0.7 % reduction compared to non-ICS users.

In spite of these measurable effects of ICS on the linear growth, it is important to recall that the safety profile of all ICS preparations, which focus anti-inflammatory effects on the lung, is markedly better than the oral glucocorticoids [130]. Published data support growth retardation, not suppression, in adult heights of children who were treated with ICSs; however, the effect is sustained and not cumulative [146]. Furthermore, it is unknown if the reductions in growth represent a permanent effect or a temporary 1- to 2-year slowing in growth velocity. ICS induced growth suppression seems to be maximal during the first year of therapy and less pronounced in subsequent years of treatment. However, physicians prescribing ICS to children should be aware of this possibility and carefully monitor linear growth [147]. Additional studies are needed to better characterize the ICS molecule dependency of growth suppression, particularly with newer molecules (mometasone, ciclesonide).

#### Adrenal insufficiency

Adrenal suppression (AS) due to exposure of the HPA axis to exogenous glucocorticoids is the most common cause of secondary adrenal insufficiency (AI). This is a well-recognized complication of most forms of steroid therapy (e.g., oral, inhaled, parenteral, or intranasal) [148]. ICS at high doses and long duration appear to be a significant independent risk factor for AI [149, 150]. Also, changing the type of ICS or reducing the dose could potentially trigger AI [151–153]. Adrenal insufficiency may cause a spectrum of presentations varying from vague symptoms of fatigue to potentially life threatening acute adrenal crises [148]. Characteristic hyperpigmentation of the skin and orthostatic hypotension usually do not occur in secondary AI [154].

HPA deficiency and AS have been considered rare in children receiving low or medium dose ICS for a short period of time [150]. Schwartz et al. [155], noted that 14 children out of 93 cases of symptomatic AI that were reported in PubMed were in fact using a moderate dose of 500 mcg or less/day of fluticasone propionate. They, therefore, recommended that pre-adolescent children who are receiving 400 mcg or more of fluticasone propionate or equivalent per day should be quarterly tested for HPA axis suppression. A Cohort study by Smith et al. [156] determined the prevalence of HPA axis suppression to be 9.3 % of 214 children using ICS. They concluded that children on low to moderate doses of ICS were still at risk of HPA axis suppression.

Screening for adrenal suppression was recommended in children taking high dose ICS (≥500 μg/day of fluticasone propionate or equivalent; ≥400 μg/day under age 12) for more than 6 months as well as considering screening in those on medium dose (251–500 μg/day of FP or equivalent; 201–400 μg/day under age 12) when there is concomitant use of nasal and topical corticosteroids, recent or frequent short courses OCS, high level of adherence to therapy, or smaller body mass for age [150]. Other indications for screening may include patients with symptoms suggestive of AS regardless of ICS dose, concomitant use of drugs that increases the bioavailability of ICS such as ritonavir or ketoconazole, recurrent respiratory infections with slow recovery, any planned surgical procedure, unexplained hypoglycemia, gastroenteritis, chronic nausea and vomiting, dehydration, heat stress, or any condition where AI might result in acute adrenal crisis [148].

Morning serum cortisol level can be used as a screening tool and abnormal results should be confirmed with low-dose ACTH stimulation tests [150]. Scalp hair cortisol was suggested as a non-invasive biomarker of HPA suppression and a sensitive tool for monitoring adherence to ICS. Its median level was found lower in 10 asthmatic children treated with ICS as compared to a healthy control group [157]. Again, hair cortisol levels of 18 asthmatic children were two fold lower compared with the period of no ICS use [158]. Salivary cortisol is increasingly used to assess patients with suspected hypo- and hypercortisolism. Measurement of salivary cortisol and cortisone responses offers an alternative to those of serum cortisol during a synacthen test in the investigation of adrenal hypofunction due to the ease of collection and independence of binding proteins allowing for determination of the free hormone [159–161].

In case of AI, oral physiologic corticosteroid replacement therapy should be prescribed and written instructions for stress corticosteroid dosing should be provided until the adrenal suppression resolves. The use of a medical identification tag is also advisable [150].

It should be kept in mind that adding a nasal steroid to ICS could increase the risk of systemic side effects [162]. Table 8 displays some published data on ICS-induced AI in children.

**Table 8:** Some reports of ICS-induced adrenal insufficiency in children and adolescents

Reference	Methodology	Findings
Todd et al. [151]	Low dose corticotropin test	A child presented with acute adrenal crisis after shift from fluticasone 1000 μg to budesonide 800 μg/day.
Gupta et al. [234]	Serum cortisol and tetracosactrin test	800 ug/day of BDP for 6 months led to subclinical HPA-axis suppression in one out of 7 children.
Drake et al. [235]	Standard short corticotropin test	Case series of 4 children on fluticasone ≥500 μg daily who presented with adrenal crises secondary to adrenal suppression.
Dunlop et al. [152]	Standard short corticotropin test	Case report of a 5 month old infant presenting with acute adrenal crisis secondary to reducing budesonide dose.
Todd et al. [153]	Variable (Standard short corticotropin test, glucagon stimulation test, decreased serum cortisol response to critical illness)	Based on surveys of doctors in the UK, 28 cases of adrenal crises in children and in adults. AI contributed to a death in one pediatric case.
Todd et al. [236]	Variable (Standard short corticotropin test, baseline serum ACTH levels)	Case series of 3 children and one adult who had adrenal crises secondary to change of ICS.
Macdessi et al. [237]	Standard short corticotropin test	Three children had adrenal crises secondary to high dose fluticasone >500 μg daily.
Santiago et al. [238]	Standard short corticotropin test	Case report of a 7 year old child on 220 μg daily who presented with acute adrenal crisis.
Skoner et al. [139]	Serum and 12 h urinary cortisol	Effects of several examined doses of mometasone furoate on cortisol levels were similar to the placebo group.
Schwartz et al. [155]	Variable (early morning basal cortisol, standard short corticotropin test, 24 h urinary cortisol)	14 children had secondary adrenal suppression with <500 μg daily fluticasone.
Smith et al. [156]	Morning serum cortisol and low dose ACTH stimulation test	Cohort study: 43 of 214 children had low early morning serum cortisol; 20 of whom had confirmed HPA suppression with low dose ACTH stimulation testing.
Zollner et al. [239]	Variable (Fasting morning serum cortisol, basal cortisol, metyrapone testing)	91 out of 143 asthmatic children had a subclinical degree of HPA axis dysfunction.
Allen et al. [240]	24 h serum and urinary cortisol at baseline and on day 42	Inhaled fluticasone furoate/vilanterol did not affect HPA axis in adolescents or adults.
Cavkaytar et al. [241]	Morning serum cortisol and low-dose ACTH stimulation test	HPA axis suppression in 7.7 % of a group of children taking ICS even at moderate doses.

Modified from Sannarangappa and Jalleh [148]

*AI* adrenal insufficiency, *HPA-axis* hypothalamic pituitary adrenal axis, *ICS* inhaled corticosteroids

#### Effect on bone mineral accretion

Corticosteroids have adverse effects on function and survival of osteoblasts and osteocytes, and with the prolongation of osteoclast survival, induce metabolic bone disease [163]. Overall, existing data suggest that the relationship between ICS use and bone mineral density (BMD) in children is conflicting and confounded by numerous other variables [164]. The conflict is probably due to various study designs, duration of use, outcome measures, and population demographics of research trials [165].

There is some concern that prolonged treatment with high doses of ICS reduces bone mass in prepubertal asthmatic children [166–168]. In children aged 4 to 17 years, the relative risk for a non-vertebral fracture appeared to increase with larger daily doses of ICS, with a relative risk of 1.1 for an average beclomethasone dose of less than 200 μg/day, 1.2 for doses of 201–400 μg/day, and 1.3 for doses greater than 400 μg/day. However, the excess risk disappeared after adjusting for markers of asthma severity, suggesting that the observed effect might result partially from the respiratory disease rather than ICS use alone [169]. The CAMP study group reported that no significant differences in BMD were noted between budesonide, nedocromil or placebo therapy [144]. In a follow up study for a median of 7 years of children with mild to moderate asthma initially randomized into the CAMP trial, cumulative ICS use was associated with a small decrease in bone mineral accretion in males not females, but with no increased risk of osteopenia. The authors concluded that this effect on BMD is outweighed by the ability to reduce the amount of OCS used [170].

Another study of asthmatic children receiving long-term, high-dose fluticasone propionate (average 771.2 μg/d) showed no significant changes in bone metabolism or BMD compared with control subjects [171]. A similar conclusion was observed by Gregson et al. [172] in children with moderate-to-severe asthma treated with fluticasone propionate (200 μg/d) or beclomethasone dipropionate (400 μg/d) for 82 weeks. Some drugs may potentiate the effect of ICS on bone including the highly active anti-retroviral drug ritonavir [173] and the antifungal drug ketoconazole [147] due to the potential for increasing the serum levels of ICS.

Corticosteroid use and worsening airflow limitation are associated with lower Vitamin D serum levels in asthmatic patients. Vitamin D supplementation might potentiate anti-inflammatory function of corticosteroids in asthmatic patients and thereby improve asthma control [174]. A significant inverse association was reported between vitamin D levels and the use of ICS in some populations [175].

The risk of osteopenia and osteoporosis seems negligible in patients receiving low to moderate dose ICS treatment, especially in the absence of co-existent conditions that affect bone mineral accretion. Those receiving high dose ICS with intermittent systemic steroids such as patients with poorly controlled asthma are particularly vulnerable to developing ICS induced bone disease [163, 165]. Impairment of BMD as a result of HPA-axis suppression from long-term high-dose ICS use also requires further study [164]. Nutritional supplementation (e.g. calcium and vitamin D) should blunt the effects of ICS on bone mineral density. Also, ICS therapy should always aim to reach the lowest effective dose that gives asthma control [176].

#### Effect on immunity

Several studies assessed the effects of high dose ICS on cell mediated immunity by using delayed type hypersensitivity skin testing as the measure of impaired cellular immunity and did not find any impairment compared with asthmatics on medications other than ICS [177] or healthy subjects [178]. The prolonged use of low dose ICS in asthmatic children has not been shown to affect cell mediated immunity either [179]. On the other hand, some recent studies have reported the association between ICS therapy and increased risk of pneumonia in adult COPD patients [180, 181]. Also, some case reports have suggested that ICS may aggravate tuberculosis (TB) [182] A nested case–control study from Korea concluded that ICS use increases the risk of TB and that clinicians should be aware of the possibility of TB development among patients who are long-term high-dose ICS users [183].

It was suggested that combinations of drugs commonly used in asthma therapy inhibit both early pro-inflammatory cytokines and key aspects of the type I interferon pathway and may curtail excessive inflammation induced by rhinovirus infections in patients with asthma, but whether this inhibits viral clearance in vivo remains to be determined [184]. ICS therapy was also found to inhibit T-helper 17 mediated immunity which may be involved in the airway inflammation of allergic asthma in children [185].

Disseminated varicella infection and increased risk of herpes zoster were associated with asthma and systemic corticosteroid therapy in children [186, 187]. However, a retrospective cohort study revealed that varicella vaccine failure and hence chicken pox outbreaks was not associated with asthma or the use of inhaled steroids, but with the use of oral steroids [188].

#### Diabetes risk

As with oral corticosteroids, ICS have been associated with an increased risk of developing diabetes and also worsening glycemic control in patients with known diabetes [189, 190]. A large Canadian study looking at a cohort of adult patients with chronic obstructive pulmonary disease (COPD) treated with ICS found an increased risk of development and progression of diabetes, especially at high doses [191]. However, a small prospective double-blind randomized controlled trial (*n* = 12) found no difference in HbA1c values in patients with known asthma or COPD and diabetes on inhaled steroids at six weeks [192]. In children, a cross sectional study revealed a statistically significant elevation of the mean HbA1c value (5.44 ± 0.75 %) among non-diabetic children with asthma as compared to the healthy control group (5.14 ± 0.41 %). However, HbA1c levels did not correlate with the cumulative dose of ICS or time of usage [193].

Despite the paucity of studies in the pediatric age group, data extrapolated from adult studies would indicate blood glucose monitoring in diabetic children on ICS therapy. Longitudinal well controlled studies are warranted in this domain.

#### Other potential adverse effects of ICS

*Ocular complications* of glucocorticoids have been a subject of concern. Although the use of systemic corticosteroids poses an increased risk for cataract, it has been difficult to establish a similar link to ICS therapy [107]. Some studies on the effect of ICS on the eyes did not exclude subjects who had been exposed to oral corticosteroids thus confounding their results [189]. The risk of subcapsular and nuclear cataract development related to ICS use seems minimal in asthmatic children, although it may be greater in older patients. The CAMP research group studied the development of posterior subcapsular cataracts associated with long-term budesonide, and only 1 child developed evidence of cataract at the end of the 6-year study period [144]. Studies on the association of glaucoma to ICS therapy showed no evidence of a direct link or increased risk of elevation of intraocular pressure in patients after the initiation of ICS therapy. However, monitoring of ocular pressure may be necessary in patients on prolonged ICS therapy especially the elderly [107].

Oral as well as inhaled steroids cause a reduction in collagen synthesis by *the skin* [189]. Thinning of the dermis and increased bruising/purpura have been noted in patients on high dose ICS but this adverse effect is more common in adults [194, 195]. One case report suggested a link between ICS and acne but no subsequent studies have analyzed this association [196]. Hypertrichosis was reported in association with ICS therapy in children. The time between the start of ICS and the occurrence of hypertrichosis varied between one month and three years [197]. Also, hair depigmentation was reported in an Afro-Caribbean girl. The hypothetical explanation may comprise direct cytotoxic effect, changes in ground substance, vasoconstriction, mechanical effects of edema, or a dysregulation of melanogenesis [198].

It has been difficult to confirm that ICS use is associated with an increased *psychiatric morbidity* [189]. A study conducted in the Netherlands reported alterations in behavior in the pediatric population [199]. Another study in an adult population suggested that high dose ICS is negatively associated with mental well being. However, it was difficult to confirm its relation to ICS rather than asthma severity [200]. Data from the CAMP study, showed was greater improvement in the total score on the Children’s Depression Inventory in the budesonide group as compared with the placebo group (a decline of 3.2 vs. 2.2, *P* = 0.01) indicating less depression [144].

An increased frequency of *teeth* malocclusion and an open bite in children using ICS has been reported [201]. A slight increase in the risk for *gastrointestinal* ulceration, gastritis and bleeding in patients taking ICS was also reported. This was reduced on using a spacer [202].

Few cases have been reported with possible hypersensitivity reactions in children with asthma and cow’s milk allergy due to milk protein traces in inhaled corticosteroids [203–205].

The ideal inhaled corticosteroid should combine high local activity with minimal systemic adverse events. The therapeutic index of corticosteroids has been improved by inhalation devices and techniques that permit direct delivery of lower doses of steroids, thus reducing systemic exposure. Despite these advances, adverse systemic effects still exist and should be looked for [147].

## Adherence to ICS therapy

Adherence to daily inhaled corticosteroid therapy is a key determinant of asthma control. Achieving good adherence is a complex task, and may require interventions not covered in current guidelines. Poor adherence may persist in children despite a high level of concordance between medical team and parents, even in the absence of socio-economic barriers to good adherence [206]. The cost impact of achieving various levels of increase in ICS adherence levels among school aged asthmatic children (5–12 years) was recently evaluated. It was concluded that effective large-scale interventions can produce substantial cost savings from even modest increases in real-world adherence to ICS therapy among Medicaid enrolled children with asthma [207].

### Factors affecting adherence to ICS therapy

There are two forms of non-adherence; intentional and unintentional. Intentional non-adherence involves choosing not to take the medication based on the patient’s own needs, knowledge, or perception. Unintentional non-adherence can result from the complexity of the treatment regimen or understanding of the medication [208]. Intentional barriers to adherence are common; driven by illness perceptions and medication beliefs, patients and parents deliberately choose not to follow the doctor’s recommendations. Common non-intentional barriers are related to family routines, child-raising issues, and to social issues such as poverty [209].

Potential factors associated with low adherence to daily ICS within a sample of minority adolescents with persistent asthma were older age and low knowledge of ICS after adjusting for other baseline characteristics. There was an inverse relationship between age and adherence [210]. With increase in age, medication taking responsibility transfers from the parent or guardian to the adolescent [211]. Moreover, complacency with outcomes uncertainty and drive for immediate gratification over delayed benefits may contribute to non-adherence in adolescents [212].

Beliefs play a crucial role in medication adherence. Two specific medication beliefs can be distinguished namely necessities and concerns. Patients can have specific thoughts related to the necessity of their medication in maintaining their health. On the other hand, patients can also have specific concerns about the possible harmful long-term effects and dependence on their medication [213]. Necessities were positively related to self-reported adherence in a group of asthmatics from the Netherlands suggesting that it could be more important to focus on necessities than on concerns in an attempt to improve adherence [214].

Taxonomy of barriers and facilitators to long-term ICS therapy adherence was proposed. These were classified into three loci of responsibility and its corresponding domains: (1) patient (cognition; motivation, attitudes and preferences; practical implementation; and parental support); (2) patient-physician interaction (communication and patient-physician relationship); and (3) health care system (resources and services). The quality of interaction with the physician (e.g., shared decision making) and access to key health care resources (e.g., lung function testing) and services (e.g., drug insurance) appear to play crucial roles in enhancing or impeding patients’ adherence, and importantly, to modify patients’ cognition, motivation, attitudes, and behaviors [215].

In a UK-wide, cross-sectional study, unexpectedly large proportion of people with asthma experienced side effects and had strong concerns about their treatment, which impeded their adherence. There was a disparity between clinicians’ estimates of the frequency of side effects and the frequency reported by asthmatics. For instance, although 46 % of people taking ICS reported sore throat, clinicians estimated that this figure would be 10 % [216].

The level of caregiver supervision is an important factor affecting adherence to ICS therapy in children with asthma. Maternal educational status was found to affect proper ICS use and adherence in asthmatic children [217].

### Monitoring of adherence

Appropriate assessment of adherence should be done in patients with difficult-to-control asthma, before making decision about increasing the treatment including possible prescription of an expensive biological therapy [218, 219].

Self-monitoring at home (e.g. symptoms, peak expiratory flowmetry), as part of a personal management plan is encouraged. Objective measures of adherence are indeed more informative than self reporting. In an ancillary study conducted in three CAMP Clinical Centers, adherence of less than 80 % was seen in 75 % of 140 children when adherence was measured objectively but only in 6 % of children when measured by means of self-report. [220] The measurement of fractional exhaled nitric oxide (FENO) might unmask non-adherence to ICS therapy. However, in many countries, the capacity to measure FENO is unlikely to be available outside specialized centers [2].

A review of the studies using electronic adherence monitoring shows that half of them report mean adherence rates of 50 % or below and the majority report rates below 75 % [221] An observational study in 135 children, 2–12 years of age, with asthma revealed that median (interquartile range) one-year adherence to ICS, as measured by electronic devices, was 84 % (70–92 %). Fifty five children (41 %) did not achieve the pre-defined level of good adherence (≥80 %) and this was associated with poorer asthma control [206].

In a multivariate analysis conducted in 6 Asian countries, three questions related to patients’ acceptance of inhaler medicines remained significantly associated with poor adherence, after adjusting for potential confounders: “I am not sure inhaler type medicines work well” (*p* < 0.001), “Taking medicines more than once a day is inconvenient” (*p* < 0.002), and “Sometimes I skip my inhaler to use it over a longer period” (*p* < 0.001) [222].

### Potential methods to improve adherence and compliance

To date, efforts to address the problem of non-adherence in childhood asthma have had little success [223]. GINA 2016 noted that only a few adherence interventions have been studied closely in asthma: [224]

- Shared decision-making for medication and dose choice
- Inhaler reminders for missed doses
- Reduced complexity of the regimen (once versus twice daily)
- Comprehensive asthma education with home visits by asthma nurses
- Clinicians reviewing feedback on their patients’ dispensing records.

Education should highlight the importance of adherence to prescribed ICS even in the absence of symptoms, and should involve literal explanation and physical demonstration of the optimal use of inhaler devices and peak flow meters. Education should be tailored according to the socio-cultural background of the family [225].

The use of a single combination inhaler, rather than separate inhalers, is generally recommended, to maximize adherence and efficacy [2]. It was suggested that once-daily ICS therapy provides a practical therapeutic option that did not appear to jeopardize the clinical efficacy of asthma controller therapy [226]. Once-daily dosing strategy was associated with lower costs and higher level of quality-adjusted life-years (QALYs) [227].

Developing electronic monitoring device technology with reminders might be a key noninvasive resource to address poor adherence in children and adolescents [228]. The Use of an electronic monitoring device with an audiovisual reminder led to significant improvements in adherence to ICS in a group of school-aged children with asthma who attended an emergency department in Auckland, New Zealand [229]. Strong potential was found for low-cost speech recognition adherence programs integrated with an electronic health record in which speech recognition telephone calls to parents were triggered when an inhaled corticosteroid refill was due or overdue [230].

Efficacy at increasing asthma self-management skills was demonstrated using group interactive learning in the school setting [231]. On the other hand, 34 children (6–11 years old) were randomized to intervention or attention control groups for 10-weeks. The intervention arm participated in weekly coping peer group support sessions and received mp3 peer-recorded asthma messages promoting adherence. At 10 weeks, no statistical difference in objectively measured adherence could be detected between the two arms adjusting for baseline adherence (*p* = 0.929). Participants’ in both study arms self-reported adherence was significantly higher than their objectively measured adherence at week 10 [232].

Targeted parenting skills were chosen to address treatment resistance, and included nurturance and autonomy granting, use of positive reinforcement strategies, predictable routines, limit setting, problem solving, taking charge when needed, and staying calm under pressure. After the 6-month intervention, adherence with ICS increased from 72.9 % to 100.0 %, (*p* = 0.013). The percentage of children with controlled asthma increased from 0 to 62.5 % (*p* = 0.026) indicating a clinically meaningful change. Parents’ ratings at 6-months suggested that asthma-related tasks and child behaviors were less problematic and their confidence to manage asthma increased [233].

## Conclusions and unmet needs

Despite the availability of several formulations of ICS and delivery devices for treatment of childhood asthma and despite the development of evidence-based guidelines, childhood asthma control remains suboptimal.

ICS are considered the most effective drugs used to suppress airway inflammation in asthma. However, Inhaled medications are only effective if they are used properly. Therefore, choosing the appropriate delivery device and offering competent education about its proper use is mandatory to gain asthma control. It is important to realize the need for step-up therapy based on poor control and step-down at three month intervals whenever good asthma control is achieved. Physicians should recognize that preschoolers are different from older children in treatment strategies. Although ICS treatment is generally considered safe in children, the potential adverse effects related to its regular use continue to be a matter of concern. ICS therapy should always aim to reach the lowest effective dose because most adverse effects are dose-dependent. It is also more common in individuals receiving concomitant oral or nasal corticosteroids. The potential adverse effects of ICS need to be weighed against the benefit of these drugs to control persistent asthma especially that its safety profile is markedly better than oral glucocorticoids.

ICS do not offer cure to asthmatic children and discontinuation leads to deterioration of clinical control, lung function, and airway responsiveness within weeks in a proportion of patients. Therefore, adherence to daily ICS therapy is a key determinant of asthma control. This mandates that asthma education follow a repetitive pattern and involve literal explanation and physical demonstration of the optimal use of inhaler devices. Education should be tailored according to the socio-cultural background of the community.

## Abbreviations

AI: Adrenal insufficiency
AP-1: Activator protein-1
AS: Adrenal suppression
BAI: Breath actuated inhaler
BDP: Beclomethasone dipropionate
BMD: Bone mineral density
CAMP: Childhood Asthma Management Program
CBP: Cyclic adenosine monophosphate response element binding protein
COPD: Chronic obstructive pulmonary disease
DPIs: Dry powder inhalers
FENO: Fractional exhaled nitric oxide
FP: Fluticasone propionate
GINA: Global Initiative for Asthma
GRE: Glucocorticoid response elements
GRs: Glucocorticoid receptors
HAT: Histone acetyl transferase
HDAC: Histone deacetylase
HDAC2: Histone deacetylase-2
HPA: Hypothalamus pituitary axis
ICS: Inhaled corticosteroids
LABA: Long-acting beta-agonist
LTRA: Leukotriene antagonists
MAP kinase: Mitogen-activated protein kinase
MDIs: Metered dose inhalers
MF/F: Mometasone furoate/formoterol fumarate
mRNA: Messenger RNA
NF-kB: Nuclear factor-kB
OCS: Oral corticosteroids
QALYs: Quality-adjusted life-years
SABA: Short Acting beta-agonist
SIGN: Scottish Intercollegiate Guidelines Network
SiT: Single inhaler therapy
VHC: Valved holding chamber

## References

[1] Thomas M (2015). Why aren’t we doing better in asthma: time for personalised medicine?. NPJ Prim Care Respir Med.

[2] Papadopoulos NG, Arakawa H, Carlsen KH, Custovic A, Gern J, Lemanske R, Le Souef P, Mäkelä M, Roberts G, Wong G, Zar H, Akdis CA, Bacharier LB, Baraldi E, van Bever HP, de Blic J, Boner A, Burks W, Casale TB, Castro-Rodriguez JA, Chen YZ, El-Gamal YM, Everard ML, Frischer T, Geller M, Gereda J, Goh DY, Guilbert TW, Hedlin G, Heymann PW, Hong SJ, Hossny EM, Huang JL, Jackson DJ, de Jongste JC, Kalayci O, Aït-Khaled N, Kling S, Kuna P, Lau S, Ledford DK, Lee SI, Liu AH, Lockey RF, Lødrup-Carlsen K, Lötvall J, Morikawa A, Nieto A, Paramesh H, Pawankar R, Pohunek P, Pongracic J, Price D, Robertson C, Rosario N, Rossenwasser LJ, Sly PD, Stein R, Stick S, Szefler S, Taussig LM, Valovirta E, Vichyanond P, Wallace D, Weinberg E, Wennergren G, Wildhaber J, Zeiger RS (2012). International consensus on (ICON) pediatric asthma. Allergy.

[3] Global Initiative for Asthma. Pocket Guide for Asthma Management and Prevention. Updated April 2015. Available at: http://ginasthma.org/wp-content/uploads/2016/01/GINA_Pocket_2015.pdf. Accessed 30 January, 2016.

[4] de Benedictis FM, Bush A (2012). Corticosteroids in respiratory diseases in children. Am J Respir Crit Care Med.

[5] De Bosscher K, Vanden Berghe W, Haegeman G (2003). The interplay between the glucocorticoid receptor and nuclear factor-kappa B or activator protein-1: molecular mechanisms for gene repression. Endocr Rev.

[6] Adcock IM, Ito K, Barnes PJ (2004). Glucocorticoid: effects on gene transcription. Proc Am Thorac Soc.

[7] Chanez P, Bourdin A, Vachier I, Godard P, Bousquet J, Vignola AM (2004). Effects of inhaled corticosteroids on pathology in asthma and chronic obstructive pulmonary disease. Proc Am Thorac Soc.

[8] Schwiebert LM, Stellato C, Schleimer RP (1996). The epithelium as a target of glucocorticoid action in the treatment of asthma. Am J Respir Crit Care Med.

[9] Barnes PJ, Adcock IM (2003). How do corticosteroids work in asthma?. Ann Intern Med.

[10] Barnes PJ (1999). Therapeutic strategies for allergic diseases. Nature.

[11] Horvath G, Wanner A (2006). Inhaled corticosteroids: effects on the airway vasculature in bronchial asthma. Eur Respir J.

[12] Hayashi R, Wada H, Ito K, Adcock IM (2004). Effects of glucocorticoids on gene transcription. Eur J Pharmacol.

[13] Bannister AJ, Schneider R, Kouzarides T (2002). Histone methylation: dynamic or static?. Cell.

[14] Barnes PJ (2010). Inhaled corticosteroids. Pharmaceuticals.

[15] Barnes PJ (2006). How corticosteroids control inflammation. Br J Pharmacol.

[16] Imhof A, Wolffe AP (1998). Transcription: gene control by targeted histone acetylation. Curr Biol.

[17] Ito K, Barnes PJ, Adcock IM (2000). Glucocorticoid receptor recruitment of histone deacetylase 2 inhibits interleukin-1β-induced histone H4 acetylation on lysines 8 and 12. Mol Cell Biol.

[18] Reichardt HM, Tuckermann JP, Gottlicher M, Vujic M, Weih F, Angel P, Herrlich P, Schütz G (2001). Repression of inflammatory responses in the absence of DNA binding by the glucocorticoid receptor. EMBO J.

[19] Hall SE, Lim S, Witherden IR, Tetley TD, Barnes PJ, Kamal AM, Smith SF (1999). Lung type II cell and macrophage annexin I release: differential effects of two glucocorticoids. Am J Physiol.

[20] Schacke H, Schottelius A, Docke WD, Strehlke P, Jaroch S, Schmees N (2004). Dissociation of transactivation from transrepression by a selective glucocorticoid receptor agonist leads to separation of therapeutic effects from side effects. Proc Natl Acad Sci U S A.

[21] Lasa M, Brook M, Saklatvala J, Clark AR (2001). Dexamethasone destabilizes cyclooxygenase 2 mRNA by inhibiting mitogen-activated protein kinase p38. Mol Cell Biol.

[22] Lasa M, Abraham SM, Boucheron C, Saklatvala J, Clark AR (2002). Dexamethasone causes sustained expression of mitogen-activated protein kinase (MAPK) phosphatase 1 and phosphatase-mediated inhibition of MAPK p38. Mol Cell Biol.

[23] Bartholome B, Spies CM, Gaber T, Schuchmann S, Berki T, Kunkel D, Bienert M, Radbruch A, Burmester GR, Lauster R, Scheffold A, Buttgereit F (2004). Membrane glucocorticoid receptors (mGCR) are expressed in normal human peripheral blood mononuclear cells and up-regulated after in vitro stimulation and in patients with rheumatoid arthritis. FASEB J.

[24] Horvath G, Wanner A (2003). Molecular targets for steroids in airway vascular smooth muscle. Arch Physiol Biochem.

[25] Borski RJ (2000). Nongenomic membrane actions of glucocorticoids in vertebrates. Trends Endocrinol Metab.

[26] Horvath G, Lieb T, Conner GE, Salathe M, Wanner A (2001). Steroid sensitivity of norepinephrine uptake by human bronchial arterial and rabbit aortic smooth muscle cells. Am J Respir Cell Mol Biol.

[27] Kumar SD, Brieva JL, Danta I, Wanner A (2000). Transient effect of inhaled fluticasone on airway mucosal blood flow in subjects with and without asthma. Am J Respir Crit Care Med.

[28] Mendes ES, Pereira A, Danta I, Duncan RC, Wanner A (2003). Comparative bronchial vasoconstrictive efficacy of inhaled glucocorticosteroids. Eur Respir J.

[29] Schoonbrood DFM, Out TA, Lutter R, Reimert CM, Vanoverveld FJ, Jansen HM (1995). Plasma-protein leakage and local secretion of proteins assessed in sputum in asthma and COPD – the effect of inhaled corticosteroids. Clin Chim Acta.

[30] Kanazawa H, Hirata K, Yoshikawa J (2002). Involvement of vascular endothelial growth factor in exercise induced bronchoconstriction in asthmatic patients. Thorax.

[31] Nocker RE, Weller FR, Out TA, de Riemer MJ, Jansen HM, van der Zee JS (1999). A double-blind study on the effect of inhaled corticosteroids on plasma protein exudation in asthma. Am J Respir Crit Care Med.

[32] Vandegraaf EA, Out TA, Roos CM, Jansen HM (1991). Respiratory membrane-permeability and bronchial hyperreactivity in patients with stable asthma – effects of therapy with inhaled steroids. Am Rev Respir Dis.

[33] Hoshino M, Takahashi M, Takai Y, Sim J, Aoike N (2001). Inhaled corticosteroids decrease vascularity of the bronchial mucosa in patients with asthma. Clin Exp Allergy.

[34] Chetta A, Zanini A, Foresi A, Del Donno M, Castagnaro A, D’Ippolito R, Baraldo S, Testi R, Saetta M, Olivieri D (2003). Vascular component of airway remodeling in asthma is reduced by high dose of fluticasone. Am J Respir Crit Care Med.

[35] Cockcroft DW, Murdock KY (1987). Comparative effects of inhaled salbutamol, sodium cromoglycate, and beclomethasone dipropionate on allergen-induced early asthmatic responses, late asthmatic responses and increased bronchial responsiveness to histamine. J Allergy Clin Immunol.

[36] Dahl R, Johansson SA (1982). Importance of duration of treatment with inhaled budesonide on the immediate and late bronchial reaction. Eur J Respir Dis.

[37] Gotshall RW (2002). Exercise-induced bronchoconstriction. Drugs.

[38] Barnes PJ (1990). Effects of corticosteroids on airway hyperresponsiveness. Am Rev Respir Dis.

[39] Adcock IM, Stevens DA, Barnes PJ (1996). Interactions of glucocorticoids and β2-agonists. Eur Respir J.

[40] Eickelberg O, Roth M, Lorx R, Bruce V, Rudiger J, Johnson M, Block LH (1999). Ligand-independent activation of the glucocorticoid receptor by β2-adrenergic receptor agonists in primary human lung fibroblasts and vascular smooth muscle cells. J Biol Chem.

[41] Korn SH, Wouters EF, Wesseling G, Arends JW, Thunnissen FB (1998). Interaction between glucocorticoids and β2-agonists: α- and β- glucocorticoid-receptor mRNA expression in human bronchial epithelial cells. Biochem Pharmacol.

[42] Usmani OS, Maneechotesuwan K, Adcock IM, Barnes PJ (2005). Glucocorticoid receptor activation following inhaled fluticasone and salmeterol. Am J Respir Crit Care Med.

[43] Ito K, Lim S, Caramori G, Cosio B, Chung KF, Adcock IM, Barnes PJ (2002). A molecular mechanism of action of theophylline: Induction of histone deacetylase activity to decrease inflammatory gene expression. Proc Natl Acad Sci U S A.

[44] Ukena D, Harnest U, Sakalauskas R, Magyar P, Vetter N, Steffen H, Leichtl S, Rathgeb F, Keller A, Steinijans VW (1997). Comparison of addition of theophylline to inhaled steroid with doubling of the dose of inhaled steroid in asthma. Eur Respir J.

[45] Lim S, Jatakanon A, Gordon D, Macdonald C, Chung KF, Barnes PJ (2000). Comparison of high dose inhaled steroids, low dose inhaled steroids plus low dose theophylline, and low dose inhaled steroids alone in chronic asthma in general practice. Thorax.

[46] Szefler SJ, Leung DY (1997). Glucocorticoid-resistant asthma: pathogenesis and clinical implications for management. Eur Respir J.

[47] Leung DY, Bloom JW (2003). Update on glucocorticoid action and resistance. J Allergy Clin Immunol.

[48] Spahn JD, Szefler SJ, Surs W, Doherty DE, Nimmagadda SR, Leung DY (1996). A novel action of IL-13: induction of diminished monocyte glucocorticoid receptor-binding affinity. J Immunol.

[49] Corrigan CJ, Brown PH, Barnes NC, Szefler SJ, Tsai JJ, Frew AJ, Kay AB (1991). Glucocorticoid resistance in chronic asthma. Glucocorticoid pharmacokinetics, glucocorticoid receptor characteristics, and inhibition of peripheral blood T cell proliferation by glucocorticoids in vitro. Am Rev Respir Dis.

[50] Matthews JG, Ito K, Barnes PJ, Adcock IM (2004). Defective glucocorticoid receptor nuclear translocation and altered histone acetylation patterns in glucocorticoid-resistant patients. J Allergy Clin Immunol.

[51] Hamasaki Y, Kohno Y, Ebisawa M, Kondo N, Nishima S, Nishimuta T, Morikawa A, Aihara Y, Akasawa A, Adachi Y, Arakawa H, Ikebe T, Ichikawa K, Inoue T, Iwata T, Urisu A, Ohya Y, Okada K, Odajima H, Katsunuma T, Kameda M, Kurihara K, Sakamoto T, Shimojo N, Suehiro Y, Tokuyama K, Nambu M, Fujisawa T, Matsui T, Matsubara T, Mayumi M, Mochizuki H, Yamaguchi K, Yoshihara S (2014). Japanese pediatric guideline for the treatment and management of bronchial asthma 2012. Pediatr Int.

[52] Canadian Thoracic Society Asthma Committee. Diagnosis and management of asthma in preschoolers. 2015. Available from: http://www.respiratoryguidelines.ca/guideline/asthma.

[53] Canadian Thoracic Society Asthma Committee. Diagnosis and management of asthma in preschoolers, children and adults. 2012. Available from: http://www.respiratoryguidelines.ca/guideline/asthma.

[54] British Guideline on the Management of Asthma: a national clinical guideline. British Thoracic Society and Scottish Intercollegiate Guidelines Network. 2014. Available from: http://sign.ac.uk/guidelines/fulltext/141/index.html.

[55] National Asthma Council Australia, Melbourne. Australian Asthma Handbook. 2015. Available from: http://www.asthmahandbook.org.au/download-order/complete-handbook.

[56] Al-Moamary MS, Alhaider SA, Al-Hajjaj MS, Al-Ghobain MO, Idrees MM, Zeitouni MO, Al-Harbi AS, Al Dabbagh MM, Al-Matar H, Alorainy HS (2012). The Saudi initiative for asthma - 2012 update: Guidelines for the diagnosis and management of asthma in adults and children. Ann Thorac Med.

[57] South African Childhood Asthma Working group. Guideline for the management of chronic asthma in children. 2009. Available from: http://www.pulmonology.co.za/downloads/guidelines/Guideline_13.pdf.

[58] Bacharier LB, Boner A, Carlsen KH, Eigenmann PA, Frischer T, Gotz M, Helms PJ, Hunt J, Liu A, Papadopoulos N, Platts-Mills T, Pohunek P, Simons FE, Valovirta E, Wahn U, Wildhaber J, Group EPA (2008). Diagnosis and treatment of asthma in childhood: a PRACTALL consensus report. Allergy.

[59] National Asthma Education and Prevention Program, Third Expert Panel on the Diagnosis and Management of Asthma. Bethesda (MD): National Heart, Lung, and Blood Institute (US); 2007. Expert Panel Report 3: Guidelines for the Diagnosis and Management of Asthma. Available from: http://www.nhlbi.nih.gov/files/docs/guidelines/asthsumm.pdf.

[60] Indian Academy of Pediatrics. Consensus statement on the diagnosis and asthma in children. 2001. Available from: http://isaac.auckland.ac.nz/resources/Asthma-3%20_guidelines.pdf.

[61] Chauhan BF, Chartrand C, Ducharme FM (2013). Intermittent versus daily inhaled corticosteroids for persistent asthma in children and adults. Cochrane Database Syst Rev.

[62] Martinez FD, Chinchilli VM, Morgan WJ, Boehmer SJ, Lemanske RF, Mauger DT (2011). Use of beclomethasone dipropionate as rescue treatment for children with mild persistent asthma (TREXA): a randomised, double-blind, placebo-controlled trial. Lancet.

[63] Amirav I, Newhouse MT (2012). Deposition of small particles in the developing lung. Paediatr Respir Rev.

[64] van Aalderen WM, Grigg J, Guilbert TW, Roche N, Israel E, Martin RJ, Colice G, Postma DS, Hillyer EV, Burden A, Thomas V, von Ziegenweidt J, Price D (2015). Small-particle Inhaled Corticosteroid as First-line or Step-up Controller Therapy in Childhood Asthma. J Allergy Clin Immunol Pract.

[65] Ni Chroinin M, Greenstone I, Lasserson TJ, Ducharme FM. Addition of inhaled long-acting beta2-agonists to inhaled steroids as first line therapy for persistent asthma in steroid-naive adults and children. Cochrane Database System Rev. 2009;(4):CD005307. 10.1002/14651858.CD005307.pub2 PMC4170786 19821344

[66] Sorkness CA, Lemanske RF, Mauger DT, Boehmer SJ, Chinchilli VM, Martinez FD, Strunk RC, Szefler SJ, Zeiger RS, Bacharier LB, Bloomberg GR, Covar RA, Guilbert TW, Heldt G, Larsen G, Mellon MH, Morgan WJ, Moss MH, Spahn JD, Taussig LM, Childhood Asthma Research and Education Network of the National Heart, Lung, and Blood Institute (2007). Long-term comparison of 3 controller regimens for mild-moderate persistent childhood asthma: the Pediatric Asthma Controller Trial. J Allergy Clin Immunol.

[67] Szefler SJ, Baker JW, Uryniak T, Goldman M, Silkoff PE (2007). Comparative study of budesonide inhalation suspension and montelukast in young children with mild persistent asthma. J Allergy Clin Immunol.

[68] Garcia Garcia ML, Wahn U, Gilles L, Swern A, Tozzi CA, Polos P (2005). Montelukast, compared with fluticasone, for control of asthma among 6- to 14-year-old patients with mild asthma: the MOSAIC study. Pediatrics.

[69] Ostrom NK, Decotiis BA, Lincourt WR, Edwards LD, Hanson KM, Carranza Rosenzweig JR, Crim C (2005). Comparative efficacy and safety of low-dose fluticasone propionate and montelukast in children with persistent asthma. J Pediatr.

[70] Zeiger RS, Szefler SJ, Phillips BR, Schatz M, Martinez FD, Chinchilli VM (2006). Response profiles to fluticasone and montelukast in mild-to-moderate persistent childhood asthma. J Allergy Clin Immunol.

[71] Wu AC, Li L, Fung V, Kharbanda EO, Larkin EK, Vollmer WM, Butler MG, Miroshnik I, Rusinak D, Davis RL, Hartert T, Weiss ST, Lieu TA (2014). Use of leukotriene receptor antagonists are associated with a similar risk of asthma exacerbations as inhaled corticosteroids. J Allergy Clin Immunol Pract.

[72] Kew KM, Karner C, Mindus SM, Ferrara G (2013). Combination formoterol and budesonide as maintenance and reliever therapy versus combination inhaler maintenance for chronic asthma in adults and children. Cochrane Database Syst Rev.

[73] Bisgaard H, Le Roux P, Bjåmer D, Dymek A, Vermeulen JH, Hultquist C (2006). Budesonide/formoterol maintenance plus reliever therapy: a new strategy in pediatric asthma. Chest.

[74] O’Byrne PM, Bisgaard H, Godard PP, Pistolesi M, Palmqvist M, Zhu Y (2005). Budesonide/formoterol combination therapy as both maintenance and reliever medication in asthma. Am J Respir Crit Care Med.

[75] Ni Chroinin M, Lasserson TJ, Greenstone I, Ducharme FM (2009). Addition of long-acting beta-agonists to inhaled corticosteroids for chronic asthma in children. Cochrane Database Syst Rev.

[76] Vaessen-Verberne AA, van den Berg NJ, van Nierop JC, Brackel HJ, Gerrits GP, Hop WC, Duiverman EJ, COMBO Study Group (2010). Combination therapy salmeterol/fluticasone versus doubling dose of fluticasone in children with asthma. Am J Respir Crit Care Med.

[77] Chauhan BF, Ducharme FM (2012). Anti-leukotriene agents compared to inhaled corticosteroids in the management of recurrent and/or chronic asthma in adults and children. Cochrane Database Syst Rev.

[78] Lemanske RF, Mauger DT, Sorkness CA, Jackson DJ, Boehmer SJ, Martinez FD, Strunk RC, Szefler SJ, Zeiger RS, Bacharier LB, Covar RA, Guilbert TW, Larsen G, Morgan WJ, Moss MH, Spahn JD, Taussig LM, Childhood Asthma Research and Education (CARE) Network of the National Heart, Lung, and Blood Institute (2010). Childhood Asthma Research and Education (CARE) Network of the National Heart, Lung, and Blood Institute. Step-up therapy for children with uncontrolled asthma receiving inhaled corticosteroids. N Engl J Med.

[79] Chauhan BF, Ducharme FM (2014). Addition to inhaled corticosteroids of long-acting beta2-agonists versus anti-leukotrienes for chronic asthma. Cochrane Database Syst Rev.

[80] Foresi A, Morelli MC, Catena E (2000). Low-dose budesonide with the addition of an increased dose during exacerbations is effective in long-term asthma control. Chest.

[81] Oborne J, Mortimer K, Hubbard RB, Tattersfield AE, Harrison TW (2009). Quadrupling the dose of inhaled corticosteroid to prevent asthma exacerbations: A randomized, double-blind, placebo-controlled, parallel-group clinical trial. Am J Respir Crit Care Med.

[82] Quon BS, Fitzgerald JM, Lemière C, Shahidi N, Ducharme FM (2010). Increased versus stable doses of inhaled corticosteroids for exacerbations of chronic asthma in adults and children. Cochrane Database Syst Rev.

[83] Garrett J, Williams S, Wong C, Holdaway D (1998). Treatment of acute asthmatic exacerbations with an increased dose of inhaled steroid. Arch Dis Child.

[84] Wilson NM, Silverman M (1990). Treatment of acute, episodic asthma in preschool children using intermittent high dose inhaled steroids at home. Arch Dis Child.

[85] Svedmyr J, Nyberg E, Thunqvist P, Asbrink-Nilsson E, Hedlin G (1999). Prophylactic intermittent treatment with inhaled corticosteroids of asthma exacerbations due to airway infections in toddlers. Acta Paediatr.

[86] Yousef E, Hossain J, Mannan S, Skorpinski E, McGeady S (2012). Early intervention with high-dose inhaled corticosteroids for control of acute asthma exacerbations at home and improved outcomes: a randomized controlled trial. Allergy Asthma Proc.

[87] Chong J, Haran C, Chauhan BF, Asher I (2015). Intermittent inhaled corticosteroid therapy versus placebo for persistent asthma in children and adults. Cochrane Database Syst Rev.

[88] Edmonds ML, Milan SJ, Camargo CA, Pollack CV, Rowe BH (2012). Early use of inhaled corticosteroids in the emergency department treatment of acute asthma. Cochrane Database Syst Rev.

[89] Beckhaus AA, Riutort MC, Castro-Rodriguez JA (2014). Inhaled versus systemic corticosteroids for acute asthma in children. A systematic review. Pediatr Pulmonol.

[90] Su XM, Yu N, Kong LF, Kang J (2014). Effectiveness of inhaled corticosteroids in the treatment of acute asthma in children in the emergency department: a meta-analysis. Ann Med.

[91] Amirav I, Newhouse MT, Minocchieri S, Castro-Rodriguez JA, Schüepp KG (2010). Factors that affect the efficacy of inhaled corticosteroids for infants and young children. J Allergy Clin Immunol.

[92] Everard M, Clark AR, Milner AD (1992). Drug delivery from jet nebulisers. Arch Dis Child.

[93] Brocklebank D, Wright J, Cates C (2001). Systematic review of clinical effectiveness of pressurised metered dose inhalers versus other hand held inhaler devices for delivering corticosteroids in asthma. BMJ.

[94] Cates CJ, Adams N, Bestall J (2001). Holding chambers versus nebulisers for inhaled steroids in chronic asthma. Cochrane Database Syst Rev.

[95] Grzelewska-Rzymowska I, Kroczynska-Bednarek J, Zarkovic J (2003). Comparison of the efficacy and safety of high doses of beclometasone dipropionate suspension for nebulization and beclometasone dipropionate via a metered-dose inhaler in steroid-dependent adults with moderate to severe asthma. Respir Med.

[96] Bisgaard H, Nikander K, Munch E (1998). Comparative study of budesonide as a nebulized suspension vs pressurized metered-dose inhaler in adult asthmatics. Respir Med.

[97] Dempsey OJ, Humphreys M, Coutie WJ, Lipworth BJ (2001). Relative lung delivery of fluticasone propionate via large volume spacer or nebuliser in healthy volunteers. Eur J Clin Pharmacol.

[98] Bisgaard H, Andersen JB, Bach-Mortensen N, Bertelsen A, Friis B, Koch C, Prahl P, Wichmann R, Osterballe O (1984). A clinical comparison of aerosol and powder administration of beclomethasone dipropionate in childhood asthma. Allergy.

[99] Adler LM, Anand C, Wright F, Barrett CF, McKeith D, Clark W, Maksimczyk P, Martin G, McKinnon C, Raffles A, Buck H (2001). Efficacy and tolerability of beclomethasone dipropionate delivered by a novel multidose dry powder inhaler (Clickhaler®) versus a metered-dose inhaler in children with asthma. Curr Ther Res.

[100] Agertoft L, Pedersen S, Nikander K (1999). Drug delivery from the Turbuhaler and Nebuhaler pressurized metered dose inhaler to various age groups of children with asthma. J Aerosol Med.

[101] Backman R, Baumgarten C, Sharma RK (2001). Fluticasone propionate via Diskus™ inhaler at half the microgram dose of budesonide via Turbuhaler™ inhaler. Clinical Drug Investigation.

[102] Amirav I, Mansour Y, Tiosano T, Chamny S, Chirurg S, Oren S, Grossman Z, Kahana L, Kahan E, Newhouse MT, Network I (2003). Safety of inhaled corticosteroids delivered by plastic and metal spacers. Arch Dis Child.

[103] Amirav I, Tiosano T, Chamny S, Chirurg S, Oren S, Grossman Z, Kahan E, Newhouse MT, Mansour Y, Network IPROS (2004). Comparison of efficiency and preference of metal and plastic spacers in preschool children. Ann Allergy Asthma Immunol.

[104] Brand PL, van der Baan-Slootweg OH, Heynens JW, de Vries TW, Versteegh FG, Vreuls RC, den Ouden WJ, Smit FJ (2001). Comparison of handling and acceptability of two spacer devices in young children with asthma. Acta Paediatr.

[105] Ammari WG, Toor S, Chetcuti P, Stephenson J, Chrystyn H (2015). Evaluation of asthma control, parents’ quality of life and preference between Aero Chamber Plus and AeroChamber Plus Flow-Vu spacers in young children with asthma. J Asthma.

[106] Dhar R, Salvi S, Rajan S, Dalal S, Tikkiwal S, Bhagat R, Ahmed MM, Balki A, Jain M, Gogtay J (2015). Salmeterol/fluticasone through breath-actuated inhaler versus pMDI: A randomized, double-blind, 12 weeks study. J Asthma.

[107] Dahl R (2006). Systemic side effects of inhaled corticosteroids in patients with asthma. Respir Med.

[108] Buhl R (2006). Local oropharyngeal side effects of inhaled corticosteroids in patients with asthma. Allergy.

[109] Roland NJ, Bhalla RK, Earis J (2004). The local side effects of inhaled corticosteroids: current understanding and review of the literature. Chest.

[110] Pinto CR, Almeida NR, Marques TS, Yamamura LL, Costa LA, Souza-Machado A (2013). Local adverse effects associated with the use of inhaled corticosteroids in patients with moderate or severe asthma. J Bras Pneumol.

[111] Barnes PJ (1995). Inhaled glucocorticoids for asthma. N Engl J Med.

[112] Godara N, Godara R, Khullar M (2011). Impact of inhalation therapy on oral health. Lung India.

[113] Rachelefsky GS, Liao Y, Faruqi R (2007). Impact of inhaled corticosteroid-induced oropharyngeal adverse events: results from a meta-analysis. Ann Allergy Asthma Immunol.

[114] Pingeleton WW, Bone RC, Kerby GR, Ruth WE (1977). Oropharyngeal candidiasis in patients treated with triamcinolone acetonide aerosol. J Allergy Clin Immunol.

[115] van Boven JF, de Jong-van den Berg LT, Vegter S (2013). Inhaled corticosteroids and the occurrence of oral candidiasis: a prescription sequence symmetry analysis. Drug Saf.

[116] Glavey SV, Keane N, Power M, O’Regan AW (2013). Posterior pharyngeal candidiasis in the absence of clinically overt oral involvement: a cross-sectional study. Lung.

[117] Fukushima C, Matsuse H, Tomari S, Obase Y, Miyazaki Y, Shimoda T, Kohno S (2003). Oral candidiasis associated with inhaled corticosteroid use: comparison of fluticasone and beclomethasone. Ann Allergy Asthma Immunol.

[118] Aun MV, Ribeiro MR, Costa Garcia CL, Agondi RC, Kalil J, Giavina-Bianchi P (2009). Esophageal candidiasis - an adverse effect of inhaled corticosteroids therapy. J Asthma.

[119] Hasosah MY, Showail M, Al-Sahafi A, Satti M, Jacobson K (2009). Esophageal candidiasis in an immunocompetent girl. World J Pediatr.

[120] Kanda N, Yasuba H, Takahashi T, Mizuhara Y, Yamazaki S, Imada Y, Izumi Y, Kobayashi Y, Yamashita K, Kita H, Tamada T, Chiba T (2003). Prevalence of esophageal candidiasis among patients treated with inhaled fluticasone propionate. Am J Gastroenterol.

[121] Mullaoglu S, Turktas H, Kokturk N, Tuncer C, Kalkanci A, Kustimur S (2007). Esophageal candidiasis and Candida colonization in asthma patients on inhaled steroids. Allergy Asthma Proc.

[122] Galván CA, Guarderas JC (2012). Practical considerations for dysphonia caused by inhaled corticosteroids. Mayo Clin Proc.

[123] Chmielewska M, Akst LM (2015). Dysphonia associated with the use of inhaled corticosteroids. Curr Opin Otolaryngol Head Neck Surg.

[124] Gallivan GJ, Gallivan KH, Gallivan HK (2007). Inhaled corticosteroids: hazardous effects on voice-an update. J Voice.

[125] Wogelius P, Poulsen S, Sorensen HT (2004). Use of asthma drugs and risk of dental caries among 5 to 7 year old Danish children: A cohort study. Community Dent Health.

[126] Milano M, Lee JY, Donovan K, Chen JW (2006). A crosssectional study of medication related factors and caries experience in asthmatic children. Pediatr Dent.

[127] Porter SR, Scully C (2006). Oral malodour (halitosis). BMJ.

[128] McDerra EJ, Bjerkeborn K, Dahllof G, Hedlin G, Lindell M, Modeer T (1988). Effect of disease severity and pharmacotherapy of asthma on oral health in asthmatic children. Scand J Dent Res.

[129] Yokoyama H, Yamamura Y, Ozeki T, Iga T, Yamada Y (2006). Influence of mouth washing procedures on the removal of drug residues following inhalation of corticosteroids. Biol Pharm Bull.

[130] Philip J (2014). The effects of inhaled corticosteroids on growth in children. Open Respir Med J.

[131] Allen DB (2002). Inhaled corticosteroid therapy for asthma in preschool children: growth issues. Pediatrics.

[132] van Bever HP, Desager KN, Lijssens N, Weyler JJ, Du Caju MVL (1999). Does treatment of asthmatic children with inhaled corticosteroids affect their adult height?. Pediatr Pulmonol.

[133] Inoue T, Doi S, Takamatsu I, Murayama N, Kameda M, Toyoshima K (1999). Effect of long-term treatment with inhaled beclomethasone dipropionate on growth of asthmatic children. J Asthma.

[134] Skoner DP, Szefler SJ, Welch M, Walton-Bowen K, Cruz-Rivera M, Smith JA (2000). Longitudinal growth in infants and young children treated with budesonide inhalation suspension for persistent asthma. J Allergy Clin Immunol.

[135] Agertoft L, Pedersen S (2000). Effect of long-term treatment with inhaled budesonide on adult height in children with asthma. New Engl J Med.

[136] Pauwels RA, Pedersen S, Busse WW, Tan WC, Chen YZ, Ohlsson SV, Ullman A, Lamm CJ, O’Byrne PM, Investigators Group START (2003). Early intervention with budesonide in mild persistent asthma: a randomized, double-blind trial. Lancet.

[137] Stefanovic IM, Verona E, Cicak B, Vrsalovic R (2011). No effect of fluticasone propionate on linear growth in preschool children with asthma. Pediatr Int.

[138] Bensch GW, Greos LS, Gawchik S, Kpamegan E, Newman (2011). Linear growth and bone maturation are unaffected by 1 year of therapy with inhaled flunisolide hydrofluoroalkane in prepubescent children with mild persistent asthma: a randomized, double-blind, placebo-controlled trial. Ann Allergy Asthma Immunol.

[139] Skoner DP, Meltzer EO, Milgrom H, Stryszak P, Teper A, Staudinger H (2011). Effects of inhaled mometasone furoate on growth velocity and adrenal function: a placebo-controlled trial in children 4–9 years old with mild persistent asthma. J Asthma.

[140] Kelly HW, Sternberg AL, Lescher R, Fuhlbrigge AL, Williams P, Zeiger RS, Raissy HH, Van Natta ML, Tonascia J, Strunk RC, CAMP Research Group (2012). Effect of inhaled glucocorticoids in childhood on adult height. N Engl J Med.

[141] Protudjer JL, Lundholm C, Bergström A, Kull I, Almqvist C (2015). The influence of childhood asthma on puberty and height in Swedish adolescents. Pediatr Allergy Immunol.

[142] Zhang L, Prietsch SO, Ducharme FM (2014). Inhaled corticosteroids in children with persistent asthma: effects on growth. Evid Based Child Health.

[143] Pruteanu AI, Chauhan BF, Zhang L, Prietsch SO, Ducharme FM (2014). Inhaled corticosteroids in children with persistent asthma: dose–response effects on growth. Evid Based Child Health.

[144] Childhood Asthma Management Program Research Group (2000). Long-term effects of budesonide or nedocromil in children with asthma. N Engl J Med.

[145] Loke YK, Blanco P, Thavarajah M, Wilson AM (2015). Impact of inhaled corticosteroids on growth in children with asthma: systematic review and meta-analysis. PLoS One.

[146] Raissy HH, Blake K (2013). Does Use of inhaled corticosteroid for management of asthma in children make them shorter adults?. Pediatr Allergy Immunol Pulmonol.

[147] Allen DB, Bielory L, Derendorf H, Dluhy R, Colice GL, Szefler SJ (2003). Inhaled corticosteroids: Past lessons and future issues. J Allergy Clin Immunol.

[148] Sannarangappa V, Jalleh R (2014). Inhaled corticosteroids and secondary adrenal insufficiency. Open Respir Med J.

[149] Lapi F, Kezouh A, Suissa S, Ernst P (2013). The use of inhaled corticosteroids and the risk of adrenal insufficiency. Eur Respir J.

[150] Issa-El-Khoury K, Kim H, Chan ES, Vander Leek T, Noya F (2015). CSACI position statement: systemic effect of inhaled corticosteroids on adrenal suppression in the management of pediatric asthma. Allergy, Asthma Clin Immunol.

[151] Todd GR, Wright D, Ryan M (1999). Acute adrenal insufficiency in a patient with asthma after changing from fluticasone propionate to budesonide. J Allergy Clin Immunol.

[152] Dunlop KA, Carson DJ, Shields MD (2002). Hypoglycemia due to adrenal suppression secondary to high-dose nebulized corticosteroid. Pediatr Pulmonol.

[153] Todd GR, Acerini CL, Ross-Russell R, Zahra S, Warner JT, McCance D (2002). Survey of adrenal crisis associated with inhaled corticosteroids in the United Kingdom. Arch Dis Child.

[154] Lovas K, Husebye ES (2005). Addison’s disease. Lancet.

[155] Schwartz RH, Neacsu O, Ascher DP, Alpan O (2012). Moderate dose inhaled corticosteroid-induced symptomatic adrenal suppression: case report and review of the literature. Clin Pediatr (Phila).

[156] Smith RW, Downey K, Gordon M, Hudak A, Meeder R, Barker S, Smith WG (2012). Prevalence of hypothalamic-pituitary-adrenal axis suppression in children treated for asthma with inhaled corticosteroid. Paediatr Child Health.

[157] Kamps AW, Molenmaker M, Kemperman R, van der Veen BS, Bocca G, Veeger NJ (2014). Children with asthma have significantly lower long-term cortisol levels in their scalp hair than healthy children. Acta Paediatr.

[158] Smy L, Shaw K, Smith A, Russell E, Van Uum S, Rieder M, Carleton B, Koren G (2015). Hair cortisol as a novel biomarker of HPA suppression by inhaled corticosteroids in children. Pediatr Res.

[159] Deutschbein T, Unger N, Mann K, Petersenn S (2009). Diagnosis of secondary adrenal insufficiency in patients with hypothalamic-pituitary disease: comparison between serum and salivary cortisol during the high-dose short synacthen test. Eur J Endocrinol.

[160] Deutschbein T, Broecker-Preuss M, Flitsch J, Jaeger A, Althoff R, Walz MK, Mann K, Petersenn S (2012). Salivary cortisol as a diagnostic tool for Cushing’s syndrome and adrenal insufficiency: improved screening by an automatic immunoassay. Eur J Endocrinol.

[161] Cornes MP, Ashby HL, Khalid Y, Buch HN, Ford C, Gama R (2015). Salivary cortisol and cortisone responses to tetracosactrin (synacthen). Ann Clin Biochem.

[162] Juniper EF, Stahl E, Doty RL, Simons FER, Allen DB, Howarth PH (2005). Clinical outcomes and adverse effect monitoring in allergic rhinitis. J Allergy Clin Immunol.

[163] Chee C, Sellahewa L, Pappachan JM (2014). Inhaled corticosteroids and bone health. Open Respir Med J.

[164] Buehring B, Viswanathan R, Binkley N, Busse W (2013). Glucocorticoid-induced osteoporosis: an update on effects and management. J Allergy Clin Immunol.

[165] Aljubran SA, Whelan GJ, Glaum MC, Lockey RF (2014). Osteoporosis in the at-risk asthmatic. Allergy.

[166] Boot AM, de Jongste JC, Verberne AA, Pols HA, de Muinck Keizer-Schrama SM (1997). Bone mineral density and bone metabolism of prepubertal children with asthma after long-term treatment with inhaled corticosteroids. Pediatr Pulmonol.

[167] Allen HD, Thong IG, Clifton-Bligh P, Holmes S, Nery L, Wilson KB (2000). Effects of high-dose inhaled corticosteroids on bone metabolism in prepubertal children with asthma. Pediatr Pulmonol.

[168] Harris M, Hauser S, Nguyen TV, Kelly PJ, Rodda C, Morton J, Freezer N, Strauss BJ, Eisman JA, Walker JL (2001). Bone mineral density in prepubertal asthmatics receiving corticosteroid treatment. J Paediatr Child Health.

[169] van Staa TP, Bishop N, Leufkens HG, Cooper C (2004). Are inhaled corticosteroids associated with an increased risk of fracture in children?. Osteoporos Int.

[170] Kelly HW, Van Natta ML, Covar RA, Tonascia J, Green RP, Strunk RC, CAMP Research Group (2008). Effect of long-term corticosteroid use on bone mineral density in children: a prospective longitudinal assessment in the childhood Asthma Management Program (CAMP) study. Pediatrics.

[171] Griffiths AL, Sim D, Strauss B, Rodda C, Armstrong D, Freezer N (2004). Effect of high-dose fluticasone propionate on bone density and metabolism in children with asthma. Pediatr Pulmonol.

[172] Gregson RK, Rao R, Murrills AJ, Taylor PA, Warner JO (1998). Effect of inhaled corticosteroids on bone mineral density in childhood asthma: comparison of fluticasone propionate with beclomethasone dipropionate. Osteoporos Int.

[173] Kaviani N, Bukberg P, Manessis A, Yen V, Young I (2011). Iatrogenic osteoporosis, bilateral HIP osteonecrosis, and secondary adrenal suppression in an HIV-infected man receiving inhaled corticosteroids and ritonavir-boosted highly active antiretroviral therapy. Endocr Pract.

[174] Searing DA, Zhang Y, Murphy JR, Hauk PJ, Goleva E, Leung DYM (2010). Decreased serum vitamin D levels in children with asthma are associated with increased corticosteroid use. J Allergy Clin Immunol.

[175] Brehm JM, Celedón JC, Soto-Quiros ME, Avila L, Hunninghake GM, Forno E, Laskey D, Sylvia JS, Hollis BW, Weiss ST, Litonjua AA (2009). Serum vitamin D levels and markers of severity of childhood asthma in Costa Rica. Am J Respir Crit Care Med.

[176] Fuhlbrigge AL, Kelly HW (2014). Inhaled corticosteroids in children: effects on bone mineral density and growth. Lancet Respir Med.

[177] Lee C, Klaustermeyer WB (2012). Effect of high dose inhaled corticosteroids on cell mediated immunity in patients with asthma. Allergol Immunopathol (Madr).

[178] England RW, Nugent JS, Grathwohl KW, Hagan L, Quinn JM (2003). High dose inhaled fluticasone and delayed hypersensitivity skin testing. Chest.

[179] Levy J, Zalkinder I, Kuperman O, Skibin A, Apte R, Bearman JE, Mielke PW, Tal A (1995). Effect of prolonged use of inhaled steroids on the cellular immunity of children with asthma. J Allergy Clin Immunol.

[180] Janson C, Larsson K, Lisspers KH, Ställberg B, Stratelis G, Goike H, Jörgensen L, Johansson G (2013). Pneumonia and pneumonia related mortality in patients with COPD treated with fixed combinations of inhaled corticosteroid and long acting β2 agonist: observational matched cohort study (PATHOS). BMJ.

[181] Suissa S, Patenaude V, Lapi F, Ernst P (2013). Inhaled corticosteroids in COPD and the risk of serious pneumonia. Thorax.

[182] Shaikh WA (1992). Pulmonary tuberculosis in patients treated with inhaled beclomethasone. Allergy.

[183] Lee CH, Kim K, Hyun MK, Jang EJ, Lee NR, Yim JJ (2013). Use of inhaled corticosteroids and the risk of tuberculosis. Thorax.

[184] Davies JM, Carroll ML, Li H, Poh AM, Kirkegard D, Towers M, Upham JW (2011). Budesonide and formoterol reduce early innate anti-viral immune responses in vitro. PLoS One.

[185] Albano GD, Di Sano C, Bonanno A, Riccobono L, Gagliardo R, Chanez P, Gjomarkaj M, Montalbano AM, Anzalone G, La Grutta S, Ricciardolo FL, Profita M (2013). Th17 immunity in children with allergic asthma and rhinitis: a pharmacological approach. PLoS One.

[186] Wi CI, Kim BS, Mehra S, Yawn BP, Park MA, Juhn YJ (2015). Risk of herpes zoster in children with asthma. Allergy Asthma Proc.

[187] Wu CT, Tsai SC, Lin JJ, Hsia SH (2008). Disseminated varicella infection in a child receiving short-term steroids for asthma. Pediatr Dermatol.

[188] Verstraeten T, Jumaan AO, Mullooly JP, Seward JF, Izurieta HS, DeStefano F, Black SB, Chen RT, Group VSDR (2003). A retrospective cohort study of the association of varicella vaccine failure with asthma, steroid use, age at vaccination, and measles-mumps-rubella vaccination. Pediatrics.

[189] Pandya D, Puttanna A, Balagopal V (2014). Systemic effects of inhaled corticosteroids: an overview. Open Respir Med J.

[190] Egbuonu F, Antonio FA, Edavalath M (2014). Effect of inhaled corticosteroids on glycemic status. Open Respir Med J.

[191] Suissa S, Kezouh A, Ernst P (2010). Inhaled corticosteroids and the risks of diabetes onset and progression. Am J Med.

[192] Faul JL, Wilson SR, Chu JW, Canfield J, Kuschner WG (2009). The effect of an inhaled corticosteroid on glucose control in type 2 diabetes. Clin Med Res.

[193] Yucel O, Eker Y, Nuhoglu C, Ceran O (2009). Hemoglobin a1c levels in children with asthma using low dose inhaled corticosteroids. Indian Pediatr.

[194] Capewell S, Reynolds S, Shuttleworth D, Edwards C, Finlay AY (1990). Purpura and dermal thinning associated with high-dose inhaled corticosteroids. BMJ.

[195] Guillot B (2002). Adverse skin reactions to inhaled corticosteroids. Expert Opin Drug Saf.

[196] Monk B, Cunliffe WJ, Layton AM, Rhodes DJ (1993). Acne induced by inhaled corticosteroids. Clin Exp Dermatol.

[197] de Vries TW, de Langen-Wouterse JJ, de Jong-Van den Berg LT, Duiverman EJ (2007). Hypertrichosis as a side effect of inhaled steroids in children. Pediatr Pulmonol.

[198] Bezzina C, Bondon-Guitton E, Montastruc JL (2013). Inhaled corticosteroid-induced hair depigmentation in a child. J Drugs Dermatol.

[199] de Vries TW, De Langden-Wouterse JJ, Van Puijenbroek E, Duiverman EJ, De Jong-Van den Berg LT (2006). Reported adverse drug reactions during the use of inhaled steroids in children with asthma in the Netherlands. Eur J Clin Pharmacol.

[200] Bonala SB, Pina D, Silverman BA, Amara S, Bassett CW, Schneider AT (2003). Asthma severity, psychiatric morbidity, and quality of life: correlation with inhaled corticosteroid dose. J Asthma.

[201] Kumar SS, Nandlal B (2012). Effects of Asthma and Inhalation corticosteroids on the dental arch morphology in children. J Indian Soc Pedod Prev Dent.

[202] Hansen RA, Tu W, Wang J, Ambuehl R, McDonald CJ, Murray MD (2008). Risk of adverse gastrointestinal events from inhaled corticosteroids. Pharmacotherapy.

[203] Nowak-Wegrzyn A, Shapiro GG, Beyer K, Bardina L, Sampson HA (2004). Contamination of dry powder inhalers for asthma with milk proteins containing lactose. J Allergy Clin Immunol.

[204] Eda A, Sugai K, Shioya H, Fujitsuka A, Ito S, Iwata T, Funabiki T (2009). Acute allergic reaction due to milk proteins contaminating lactose added to corticosteroid for injection. Allergol Int.

[205] Savvatianos S, Giavi S, Stefanaki E, Siragakis G, Manousakis E, Papadopoulos NG (2011). Cow’s milk allergy is a cause of anaphylaxis to systemic corticosteroids. Allergy.

[206] Klok T, Kaptein AA, Duiverman EJ, Brand PL (2015). Long-term adherence to inhaled corticosteroids in children with asthma: Observational study. Respir Med.

[207] Rust G, Zhang S, McRoy L, Pisu M (2015). Potential savings from increasing adherence to inhaled corticosteroid therapy in Medicaid-enrolled children. Am J Manag Care.

[208] Lee JX, Wojtczak HA, Wachter AM, Lee ML, Burns L, Chen D, Yusin JS (2015). Understanding asthma medical nonadherence in an adult and pediatric population. J Allergy Clin Immunol Pract.

[209] Klok T, Kaptein AA, Brand PL (2015). Non-adherence in children with asthma reviewed: The need for improvement of asthma care and medical education. Pediatr Allergy Immunol.

[210] Mosnaim G, Li H, Martin M, Richardson D, Belice PJ, Avery E, Ryan N, Bender B, Powell L (2014). Factors associated with levels of adherence to inhaled corticosteroids in minority adolescents with asthma. Ann Allergy Asthma Immunol.

[211] Orrell-Valente JK, Jarslberg LG, Hill LG, Cabana MD (2008). At what age do children start taking daily asthma medicines on their own?. Pediatrics.

[212] Brandt S, Dickinson B (2013). Time and risk preferences and the use of asthma controller medication. Pediatrics.

[213] Horne R, Weinman J (1999). Patients’ beliefs about prescribed medicines and their role in adherence to treatment in chronic physical illness. J Psychosom Res.

[214] Van Steenis M, Driesenaar J, Bensing J, Van Hulten R, Souverein P, Van Dijk L, De Smet P, Van Dulmen A (2014). Relationship between medication beliefs, self-reported and refill adherence, and symptoms in patients with asthma using inhaled corticosteroids. Patient Prefer Adherence.

[215] Peláez S, Lamontagne AJ, Collin J, Gauthier A, Grad RM, Blais L, Lavoie KL, Bacon SL, Ernst P, Guay H, McKinney ML, Ducharme FM (2015). Patients’ perspective of barriers and facilitators to taking long-term controller medication for asthma: a novel taxonomy. BMC Pulm Med.

[216] Cooper V, Metcalf L, Versnel J, Upton J, Walker S, Horne R (2015). Patient-reported side effects, concerns and adherence to corticosteroid treatment for asthma, and comparison with physician estimates of side-effect prevalence: a UK-wide, cross-sectional study. NPJ Prim Care Respir Med.

[217] Capanoglu M, Dibek Misirlioglu E, Toyran M, Civelek E, Kocabas CN (2015). Evaluation of inhaler technique, adherence to therapy and their effect on disease control among children with asthma using metered dose or dry powder inhalers. J Asthma.

[218] Gamble J, Stevenson M, McClean E, Heaney LG (2009). The prevalence of nonadherence in difficult asthma. Am J Respir Crit Care Med.

[219] Heaney LG, Horne R (2012). Non-adherence in difficult asthma: time to take it seriously. Thorax.

[220] Krishnan JA, Bender BG, Wamboldt FS, Szefler SJ, Adkinson NF, Zeiger RS, Wise RA, Bilderback AL, Rand CS, Adherence Ancillary Study Group (2012). Adherence to inhaled corticosteroids: an ancillary study of the Childhood Asthma Management Program clinical trial. J Allergy Clin Immunol.

[221] Morton RW, Everard ML, Elphick HE (2014). Adherence in childhood asthma: the elephant in the room. Arch Dis Child.

[222] Chiu KC, Boonsawat W, Cho SH, Cho YJ, Hsu JY, Liam CK, Muttalif AR, Nguyen HD, Nguyen VN, Wang C, Kwon N (2014). Patients’ beliefs and behaviors related to treatment adherence in patients with asthma requiring maintenance treatment in Asia. J Asthma.

[223] Osterberg L, Blaschke T (2005). Adherence to medication. N Engl J Med.

[224] Global Initiative for Asthma. Pocket Guide for Asthma Management and Prevention. Updated April 2016. Available from: http://ginasthma.org/2016-pocket-guide-for-asthma-management-and-prevention/. Accessed 25 June 2016.

[225] Andrade WC, Camargos P, Lasmar L, Bousquet J (2010). A pediatric asthma management program in a low-income setting resulting in reduced use of health service for acute asthma. Allergy.

[226] Wells KE, Peterson EL, Ahmedani BK, Williams LK (2013). Real-world effects of once s greater daily inhaled corticosteroid dosing on medication adherence. Ann Allergy Asthma Immunol.

[227] Rodriguez-Martinez CE, Sossa-Briceño MP, Castro-Rodriguez JA (2016). Cost-utility analysis of once-daily versus twice-daily inhaled corticosteroid dosing for maintenance treatment of asthma in pediatric patients. J Asthma.

[228] Anderson WC, Szefler SJ (2015). New and future strategies to improve asthma control in children. J Allergy Clin Immunol.

[229] Chan AH, Stewart AW, Harrison J, Camargo CA, Black PN, Mitchell EA (2015). The effect of an electronic monitoring device with audiovisual reminder function on adherence to inhaled corticosteroids and school attendance in children with asthma: a randomised controlled trial. Lancet Respir Med.

[230] Bender BG, Cvietusa PJ, Goodrich GK, Lowe R, Nuanes HA, Rand C, Shetterly S, Tacinas C, Vollmer WM, Wagner N, Wamboldt FS, Xu S, Magid DJ (2015). Pragmatic trial of health care technologies to improve adherence to pediatric asthma treatment: a randomized clinical trial. JAMA Pediatr.

[231] Mosnaim GS, Pappalardo AA, Resnick SE, Codispoti CD, Bandi S, Nackers L, Malik RN, Vijayaraghavan V, Lynch EB, Powell LH (2016). Behavioral Interventions to Improve Asthma Outcomes for Adolescents: A Systematic Review. J Allergy Clin Immunol Pract.

[232] Mosnaim G, Li H, Martin M, Richardson D, Belice PJ, Avery E, Ryan N, Bender B, Powell L (2013). The impact of peer support and mp3 messaging on adherence to inhaled corticosteroids in minority adolescents with asthma: a randomized, controlled trial. J Allergy Clin Immunol Pract.

[233] Garbutt JM, Sylvia S, Rook S, Schmandt M, Ruby-Ziegler C, Luby J, Strunk RC (2015). Peer training to improve parenting and childhood asthma management skills: a pilot study. Ann Allergy Asthma Immunol.

[234] Gupta D, Behera D, Lalrinmawia H, Dash RJ (2000). Hypothalamo-pituitary-adrenal axis function in asthmatics taking low dose inhaled beclomethasone dipropionate. J Assoc Physicians India.

[235] Drake AJ, Howells RJ, Shield JP, Prendiville A, Ward PS, Crowne EC (2002). Symptomatic adrenal insufficiency presenting with hypoglycaemia in children with asthma receiving high dose inhaled fluticasone propionate. BMJ.

[236] Todd GR, Acerini CL, Buck JJ, Murphy NP, Ross-Russell R, Warner JT, McCance DR (2002). Acute adrenal crisis in asthmatics treated with high-dose fluticasone propionate. Eur Respir J.

[237] Macdessi JS, Randell TL, Donaghue KC, Ambler GR, van Asperen PP, Mellis CM (2003). Adrenal crises in children treated with high-dose inhaled corticosteroids for asthma. Med J Aust.

[238] Santiago AH, Ratzan S. Acute adrenal crisis in an asthmatic child treated with inhaled fluticasone proprionate. Int J Pediatr Endocrinol. 2010;2010. 10.1155/2010/749239 PMC2931373 20814595

[239] Zollner EW, Lombard CJ, Galal U, Hough FS, Irusen EM, Weinberg E (2012). Hypothalamic-pituitary-adrenal axis suppression in asthmatic school children. Pediatrics.

[240] Allen A, Schenkenberger I, Trivedi R, Cole J, Hicks W, Gul N, Jacques L (2013). Inhaled fluticasone furoate/vilanterol does not affect hypothalamic-pituitary-adrenal axis function in adolescent and adult asthma: randomised, double-blind, placebo-controlled study. Clin Respir J.

[241] Cavkaytar O, Vuralli D, Arik Yilmaz E, Buyuktiryaki B, Soyer O, Sahiner UM, Kandemir N, Sekerel BE (2015). Evidence of hypothalamic-pituitary-adrenal axis suppression during moderate-to-high-dose inhaled corticosteroid use. Eur J Pediatr.

